# Immunogenicity of PE18, PE31, and PPE26 proteins from *Mycobacterium tuberculosis* in humans and mice

**DOI:** 10.3389/fimmu.2023.1307429

**Published:** 2023-12-06

**Authors:** María García-Bengoa, Emil Joseph Vergara, Andy C. Tran, Lorenzo Bossi, Andrea M. Cooper, John E. Pearl, Tufária Mussá, Maren von Köckritz-Blickwede, Mahavir Singh, Rajko Reljic

**Affiliations:** ^1^ Institute of Biochemistry, University of Veterinary Medicine Hannover, Hannover, Germany; ^2^ Research Center for Emerging Infections and Zoonosis (RIZ), University of Veterinary Medicine Hannover, Hannover, Germany; ^3^ LIONEX Diagnostics and Therapeutics GmbH, Braunschweig, Germany; ^4^ Institute for Infection and Immunity, St. George’s University of London, London, United Kingdom; ^5^ Immunxperts SA, a Q² Solutions Company, Gosselies, Belgium; ^6^ Department of Infection, Immunity and Inflammation, University of Leicester, Leicester, United Kingdom; ^7^ Department of Microbiology, Faculty of Medicine, Eduardo Mondlane University, Maputo, Mozambique

**Keywords:** tuberculosis, PE18, PE31, PPE26, antigens, vaccine, immunity

## Abstract

**Introduction:**

The large family of PE and PPE proteins accounts for as much as 10% of the genome of *Mycobacterium tuberculosis*. In this study, we explored the immunogenicity of three proteins from this family, PE18, PE31, and PPE26, in humans and mice.

**Methods:**

The investigation involved analyzing the immunoreactivity of the selected proteins using sera from TB patients, IGRA-positive household contacts, and IGRA-negative BCG vaccinated healthy donors from the TB endemic country Mozambique. Antigen-recall responses were examined in PBMC from these groups, including the evaluation of cellular responses in healthy unexposed individuals. Moreover, systemic priming and intranasal boosting with each protein, combined with the Quil-A adjuvant, were conducted in mice.

**Results:**

We found that all three proteins are immunoreactive with sera from TB patients, IGRA-positive household contacts, and IGRA-negative BCG vaccinated healthy controls. Likewise, antigen-recall responses were induced in PBMC from all groups, and the proteins stimulated proliferation of peripheral blood mononuclear cells from healthy unexposed individuals. In mice, all three antigens induced IgG antibody responses in sera and predominantly IgG, rather than IgA, responses in bronchoalveolar lavage. Additionally, CD4+ and CD8+ effector memory T cell responses were observed in the spleen, with PE18 demonstrating the ability to induce tissue-resident memory T cells in the lungs.

**Discussion:**

Having demonstrated immunogenicity in both humans and mice, the protective capacity of these antigens was evaluated by challenging immunized mice with low-dose aerosol of *Mycobacterium tuberculosis* H37Rv. The *in vitro* Mycobacterial Growth Inhibition Assay (MGIA) and assessment of viable bacteria in the lung did not demonstrate any ability of the vaccination protocol to reduce bacterial growth. We therefore concluded that these three specific PE/PPE proteins, while immunogenic in both humans and mice, were unable to confer protective immunity under these conditions.

## Introduction

Tuberculosis (TB) is a contagious infectious disease caused by *Mycobacterium tuberculosis* (Mtb). TB remains a significant public health issue globally, with nearly one-quarter of the world’s population infected with Mtb, and around 10.6 million new cases with 1.6 million deaths in 2021 (1). The rise of drug-resistant strains of Mtb has further complicated treatment efforts. Additionally, the *Bacillus Calmette-Guérin* (BCG) vaccine, the only licensed TB vaccine, although providing partial protection against disseminated forms of TB in children, has limited efficacy in preventing pulmonary TB in adults (1–3). Furthermore, BCG appears to be less effective in areas of the world with higher prevalence of exposure to environmental mycobacteria also known as non-tuberculous mycobacteria (NTM), such as the South-East Asian and the African Regions. The working model is that since some of these bacteria share numerous antigens with Mtb, including the proteins from the distinctive Proline-glutamic (PE)/proline-proline-glutamic (PPE) family (4, 5), the exposure to NTM alters the immune response to these potentially cross-reactive proteins. These issues highlight the urgent need for novel vaccines that can provide better protection against TB.

PE and PPEs are unique to mycobacteria (4, 6, 7) and abundantly expressed by Mtb, making up to 10% of the total Mtb genome (6). In recent years, these proteins have garnered significant interest due to their unique characteristics, role in virulence, and antigenic potential (8–10). Specifically, two subunit vaccines containing PPE proteins in their composition, the M72/AS01E (GlaxoSmith-Kline) and the ID93-GLASE (IDRI) vaccines, which are in phase IIb and I human clinical trials respectively. The two vaccines elicit both humoral and cellular immune responses with M72/AS01E able to increase the frequency of antigen-specific CD4+ T cells in both HIV-positive and -negative adults in a phase II randomized controlled trial in India (10, 11), and the IDRI vaccine able to elicit specific humoral and cellular responses in 60 non-BCG vaccinated healthy individuals (12). Importantly, M72/AS01E also conferred about 50% protection from active TB in pre-exposed (latent) individuals for at least the first three years (11). These studies highlight the potential of PE/PPE proteins as immunogenic targets and their ability to be part of a vaccine capable of inducing protective immune responses to TB.

The ESX5 secretion system of Mtb is responsible for the transport and secretion of a subset of PE and PPE proteins (13). However, the precise roles of the majority of these proteins in the pathogenesis of Mtb, host-pathogen interactions, and immunity, are still being elucidated. One of these proteins, PPE26, binds to TLR2 in RAW264.7 macrophages, leading to the induction of Th1-type immune responses in C57BL/6 immunized mice. This was demonstrated by the polarization of naïve CD4+ T cells, resulting in increased CXCR3 expression and secretion of IFNγ and IL-2 (14). Further, immunization of C57BL/6 mice with rBCG::PPE26 induced proliferation of effector memory CD4+/CD8+ T cells (14). Other studies have shown some protection conferred by PPE26 in immunised mice (15, 16). Thus, there is evidence supporting the hypothesis that PPE26 is an attractive candidate for TB vaccine development.

The host immune response against Mtb infection is thought to be primarily driven by Th1 CD4+ and CD8+ mediated immunity (17, 18). PE proteins, like PE9 or PE_PGRS42, seem to be potential targets for CD8+ T cell responses, suggesting that PEs may influence cellular immune responses in Mtb infection (19). Previously, the immunogenic potential of PE18 has been suggested (20, 21). Furthermore, PE18 of Mtb shares ~90% homology with a PE protein from the NTM *Mycobacterium avium* (MaPE). MaPE induced increased levels of IFNγ by both CD4+ and CD8+ T cells, but no sera antibodies in a mouse model of TB (5). In addition, in the same study, immunization with a MaPE-DNA vaccine limited the replication of Mtb in the spleens and lungs from C57BL/6 mice challenged with a low-dose of Mtb Erdman, suggesting that mycobacteria express PE antigens with cross-protective T cell epitopes in TB (5). This suggests that PE18 could potentially elicit immune responses that cross-react with MaPE, or even with similar PE proteins of Mtb or other mycobacterial strains. Furthermore, the shared homology between MaPE and PE18 raises intriguing possibilities about the presence of analogous PE/PPE antigens, like PE18, in a range of mycobacterial species. This includes NTM as well as BCG strains. Importantly, the presence of *pe* and *ppe* genes can be variable across different BCG strains (22, 23). For instance, certain BCG strains might exhibit the absence or downregulation of genes such as those encoding proteins PPE26 and PE18, which contrasts with their overexpression in the BCG Tice strain (24). This overexpression can be attributed to the duplication of the ESX5 locus (24) within this specific BCG strain. Thus, a clear understanding of the presence of specific PE/PPE antigens across different mycobacterial species requires more comprehensive genomic and proteomic investigations. Similarly, Mtb PE31 is an important virulence factor (25), and exhibits potent immunogenic properties (26). Myllymäki et al. observed that PE31 from *Mycobacterium marinum* shares 89% homology with Mtb-PE31 (an ESX5-associated protein) (26). Notably, *M. marinum* PE31 was found to induce a protective response against a low dose mycobacterial challenge in that study (26). Furthermore, given the high homology between Mtb PE18 and PE31, it is plausible that these two *pe* genes could exert similar functions. Significantly, peptides of PPE26, PE31 and PE18, all present in both Mtb H37Rv and *M. bovis*, were shown to produce IFNγ responses in both TB patients and cattle infected with *M. bovis*, reflecting a similar immune recognition hierarchy independent of the stage of disease (27). Overall, the high sequence diversity and shared structural features observed among PE and PPE proteins suggest the possibility of general cross-reactivity, and this may also apply to PPE26, PE18 and PE31. Hence, these three antigens were selected for our study.

In this study, we analysed the immunogenic potential of PE18 (Rv1788), PPE26 (Rv1789), and PE31 (Rv3477) proteins of Mtb in humans and in immunised mice. To do so, respective proteins were produced in recombinant form as described before (28), and tested for immunoreactivity with sera and PBMC from TB patients, IGRA^+^ household contacts, and IGRA^-^ BCG vaccinated healthy controls from the TB endemic country, Mozambique. We then tested their immunogenicity and protective potential in C57BL/6 mice after aerosol challenge with a low dose of *Mycobacterium tuberculosis* H37Rv.

## Materials and methods

### Expression and purification of recombinant proteins

The purification of the recombinant proteins was carried out as described before (28). Briefly, the plasmids containing genes of interest fused to 6xHis-tags were transformed and expressed in *E. coli BL21(DE3)* strain and the recombinant proteins were later purified by metal ion affinity chromatography. The individual proteins PE18 (Rv1788), PE31 (Rv3478) and PPE26 (Rv1789) were purified by the on-column refolding method using an 8M Urea gradient. Purity was assessed by SDS-PAGE (>97% purity) and identity confirmed by Western blot. Molecular weight references for SDS-PAGE and Western blot were provided using PageRuler Unstained Protein Ladder (Thermofisher, 26614) and PageRuler Prestained Protein Ladder (Thermofisher, 26616), respectively. The concentration of the recombinant proteins was determined using the Lowry assay (BioRad Laboratories, Inc.) and the endotoxin content determined using Limulus amebocyte lysate (LAL) test (PYROTELL^®^-T Lysate).

### Study population

Peripheral blood was obtained from a cohort of TB patients (EMI-TB Verification Cohort, Maputo Region, Mozambique) under the framework of the EMI-TB project. Briefly, patients with TB were confirmed by tuberculin skin test (TST) and/or the Quantiferon-TB-Gold test (QST), radiographic examination was used to discriminate between LTBI and ATB, and observation of acid-fast bacilli in sputum by Lowenstein-Jensen and Colletsos culture was used to confirm all TB-diagnosed patients. Exclusion criteria were age <18 years, co-infection with the human immunodeficiency virus (HIV) and any other immunosuppressive medical condition or concomitant use of immunosuppressive drugs. The participants in this study were originally part of the wider EMI-TB project. For the current study, we had access to a subset of serum samples and PBMCs. Sera and PBMC samples were collected after final diagnosis and before commencement of the anti-TB drug treatment. Study groups were formed as follows: ATB (n=16), LTBI (n=19) and Healthy Controls (HC, BCG vaccinated volunteers with no evidence of TB; n=17). The study was approved by the Mozambican National Bioethics committee (IRB:00002657; ID: 298/CNBS/15).

### Animals

Female C57BL/6 mice at the age of 6–8 weeks were obtained from Charles River (UK) and bred in-house at University of Leicester Preclinical Research Facility. The mice were then divided in five groups (n=10 for PBS and BCG, n=9 for PE/PPE vaccinated), with 3 animals in each group assigned for immunogenicity analysis and the remaining for Mtb challenge. All animals were used with approval from University of Leicester Ethics Committee under an approved UK Home Office animal project license (Establishment License X1798C4D2) and used in accordance with the Animals (Scientific Procedures) Act 1986.

### Immunization of C57BL/6 mice

C57BL/6 mice were immunized subcutaneously in the flank with 10µg of each PE/PPE protein and 1µg Quil-A (Invivogen) or a matched volume of saline solution (Sigma) as a control. Quil-A, known for inducing a robust adjuvant response, promotes Th1-biased immune responses and potentiates the response to mucosal antigens (29). A second subcutaneous immunization was administered three weeks later in the opposing flank, followed by a final intranasal boosting under isoflurane anaesthesia (using 0.1µg Quil-A per animal in this case) three weeks after. BCG vaccinated mice were given 5×10^5^ CFU BCG Pasteur in 0.1mL subcutaneously in the first week. Three weeks after the last immunization, mice from each group (n=3) were humanely sacrificed for immunogenicity analysis by overdose of pentobarbital given intraperitoneally, and death was verified by severing femoral artery. At the same time, remaining mice (n=6 from each group for PE/PPE and n=7 for PBS and BCG) were used for an Mtb challenge experiment. Further details of the immunization and dosing regimen are given in the corresponding figure legends.

### Challenge with M. tuberculosis

The Mtb strain H37Rv was grown in Proskauer Beck medium containing 0.05% Tween-80 to mid-log phase and frozen in 1mL aliquots at -70°C. In containment level 3 facilities, mice were challenged with approximately 100 colony forming units (CFU) using a self-contained bespoke aerosol chamber (Walker Safety Cabinets Ltd., Glossop, UK) based on the “jet in air” venturi nebulizer. On day 1 (for determination of infectious dose) and three weeks after Mtb challenge, animals were euthanised by overdose of pentobarbital given intraperitoneally, and lungs were aseptically collected to evaluate mycobacterial burden.

### Preparation of tissues from mice

#### Isolation of splenocytes

The spleens of immunised, uninfected mice were collected in 1mL of MACS Tissue preservation media (Miltenyi Biotec) and rinsed in PBS. Afterwards, spleens were mechanically disrupted through a 40µm Corning^®^ cell strainer (Sigma-Aldrich) prewashed with 2mL of cold R10 media (RPMI, 10% FBS, 5mM L-Glutamine, 100units/ml penicillin, 100µg/ml streptomycin, 10mM HEPES and 50µM 2-β-mercaptoethanol) (Sigma) and the cells were further washed with 18mL R10. Cells were centrifuged at 250g for 5 minutes at room temperature and resultant pellets were lysed with ammonium–chloride–potassium (ACK) lysis buffer (Sigma-Aldrich) to remove red blood cells for additional 5 minutes after which the reaction was stopped by addition of R10 media. Cells were maintained in complete RPMI medium in a humidified incubator at 37°C and 5% CO_2_. Spleens were processed for splenocyte re-stimulation assays to assess antigen-specific cell-mediated responses to PE/PPE antigens.

#### Isolation of lung cells

Lungs were collected in Petri dishes containing 1mL of MACS Tissue preservation media (Miltenyi Biotec) and rinsed in sterile PBS. Lungs were cut into 1mm sections and resuspended in 1mL D-PBS. Afterwards, cells were incubated for 40 minutes at 37°C with constant agitation with 1mL of 2x digestion buffer D-PBS with 1mg/mL collagenase and 0.15mg/mL DNaseI, and finally dissociated by slow pipetting. Cells were then passed through a 70µm cell strainer Corning^®^, after which R10 medium was added to quench the enzyme activity and cells were centrifuged at 250g for 5 minutes at room temperature, and lysed with ACK lysis buffer (Sigma-Aldrich) for 5 minutes. The lysis was stopped through the addition of R10 medium. Cells were centrifuged again at 250g for 5 minutes and resuspended in complete RPMI medium. The lung single cell suspensions were used to assess percentage of tissue resident memory T cells.

#### Preparation of sera from mice

Blood was withdrawn by cardiac puncture for serological analysis and allowed to clot at room temperature for 1h followed by centrifugation at 1000-2000g for 10 min at 4°C. Sera were stored at -20°C until analysis.

#### Collection of bronchoalveolar lavage fluid

Bronchoalveolar lavage was collected from lungs of vaccinated sacrificed animals by injecting 1mL of sterile PBS into the lungs via an incision in the trachea followed by three rounds of flushing. Afterwards, the washes were centrifuged at 1000g and supernatant was collected and stored at −20°C until further use.

#### Bacterial enumeration in mouse lungs

Bacterial burden from mouse organs was assessed by CFU enumeration. Lung homogenates were prepared by GentleMACS Dissociator (Myltenyi Biotec) in solution containing 0.1% Triton X-100. Homogenates were plated in technical duplicates on Middlebrooks 7H11 agar (BD Biosciences) supplemented with oleic acid-albumin-dextrose-catalase (OADC) (Millipore), glycerol and Selectab (Mast Diagnostics). CFUs were counted after 3–4 weeks incubation at 37°C.

### Splenocyte re-stimulation with PE/PPE antigens and BCG whole cell lysates

Cells from splenocytes of unimmunized, BCG immunized, and PE/PPE-immunized mice were quantified using the TC20™ automated cell counter (BioRad Laboratories, Inc.).

For antigen-recall experiments, splenocytes were incubated with respective antigens or controls (PBS or BCG whole cell lysate) in 96-well flat-bottom plates in R10 medium for 96h at 37°C with 5% CO_2_ in a humidified atmosphere. One million cells per well were used and samples were plated in duplicates. Eight hours before harvesting the cells, a combination of 50ng/mL PMA and 500ng/mL Ionomycin was added to respective wells as positive control. Between 5-6h before collecting the cells, 5µg/mL brefeldin A was added to each well for 4h to block cell trafficking. Cells were harvested and stained for surface and intracellular markers followed by flow cytometry analysis. Resultant supernatants were collected after centrifugation and stored at -80°C for cytokine ELISA.

### ELISA analysis of cytokines from splenocyte re-stimulation assay

For the assessment of cytokine production, supernatants from the re-stimulation assay were collected and used for quantitative ELISA to measure concentrations of IFNγ (Invitrogen 88-7314-88), TNF (Invitrogen 88-7324-88), IL-4 (Invitrogen 88-7044-88), IL-10 (Invitrogen 88-7105-88), and IL-17A (Invitrogen 88-7371-88), according to manufacturer’s instructions. A 1:2 dilution was used for IFNɣ and TNF, while a 1:3 dilution was used for IL-4, IL-10 and IL-17A. Cytokine concentrations in the supernatants were determined by interpolating from standard curves, and the data were plotted using GraphPad Prism V10.

### Flow cytometry analysis of mouse cells

Single cell suspensions from 96h stimulated splenocytes were added to a 96-well U-bottom plate, washed in PBS and further incubated with Fc Block (Human TruStainFcX™/TruStain fcX*™* (anti-mouse CD16/32) for 45 minutes at 4°C together with fluorochrome-conjugated mAb anti-CD3-APC/Cy7, CD4-PerCP-Cy5.5, CD8-AF700, CD44-FITC, CD62L-PE (BioLegend, San Diego, CA, USA) and fixable viability dye-BV510 (BD Biosciences). Afterwards, cells were washed twice in PBS, and subsequently fixed/permeabilized using a Fix/Perm kit according to manufacturer instructions (Thermofischer Scientific).

Cells were then washed twice with permeabilization buffer and expression levels of intracellular TNF, IFNγ and IL-2 (Panel A) or IL-17 and IL-2 (Panel B) were analysed by flow cytometry after 1h incubation at 4°C with specific anti-mouse antibodies (Panel A: TNF-APC, IFNγ-PE-Dazzle 594, IL-2-BV605 or Panel B: IL-17-PE-Cy7 and IL-2-BV605, respectively), diluted in permeabilization buffer.

Spleen cells were gated by forward and side scatter. T effector memory (Tem) cells were gated by expression of CD3, then CD4 or CD8, and finally CD44+ and CD62L-. Flow cytometry data was obtained on a Beckman Coulter CytoFLEX cytometer (BD Biosciences). The data were processed using FlowJoTM v10.8.1 (BD Biosciences, Ashland, OR, USA). Each T cell population was analysed individually for cell proliferation and activation. Fluorescence minus one (FMO) controls were used for each marker to set the appropriate gates and determine positive populations. Examples of gating strategies are shown in **Supplementary Figure S1**.

For detection of lung resident memory T cells (Trm), 4 million mononuclear cells were plated per well in a 96-well U-bottom plate for staining of surface markers. The same protocol that was used for the spleens was also applied for the lungs, in which cocktail of antibodies for surface staining contained CD44-FITC, CD4-PerCP/Cy5.5, CD3-APC, CD62L-PE, CD69-PE-Cy7, CD103-BV421, CD8-BV510 (BioLegend, San Diego, CA, USA) and Fixable Viability dye-BV510 (BD Biosciences). Gating strategy for lung-resident memory T cells is illustrated in **Supplementary Figure S2**.

### Modified mycobacterial growth inhibition assay

A modified method of mycobacterial growth inhibition assay (MGIA) was used to assess the capacity of mouse splenocytes for *in vitro* killing of *M. tuberculosis* H37Rv-lux. Briefly, C57BL/6-M cells (Kerafast, ENH166-FP) were seeded onto 48 well plates (Corning, 3548) at a density of 5x10^5^ cells per well using antibiotic-free R10 (RPMI with 10% FBS, 5mM L-Glutamine and 10mM HEPES). Cells were then infected with H37Rv-lux using a multiplicity of infection (MOI) of 1:1. After 48 h, 3x10^6^ splenocytes were added on top of infected cells. Plates were incubated for an additional 120h in a humidified CO_2_ incubator. Afterwards, media were aspirated from each well followed by cell lysis using 200µL of sterile distilled water. Luciferase assay was done to quantify viable bacteria in each well as previously described (30). Briefly, cell lysates were added to tubes (Greiner Bio-One, 115101) with 1mL of 0.1% n-decyl aldehyde (Sigma-Aldrich, W236217-SAMPLE-K). Bioluminescence was measured using a Junior LB Portable Luminometer (Berthold Technologies) set to collect data for 30 seconds. Data are expressed as relative light units (RLU).

### Determination of PE/PPE-specific IgG and IgA isotypes by ELISA

ELISA assays were used to determine antigen-specific titres for IgG isotypes in serum and BAL of vaccinated mice, and IgA in the BAL of these animals. Microtiter 96-well plates (Nunc, Maxisorp) were coated with 5µg/mL of the respective PE/PPE protein in 0.1M NaHCO_3_ pH 9.6, at 37°C for 2h, after which the coating solution was removed and the plates washed three times with distilled water. Plates were later blocked with 5% skimmed milk powder in PBS for another 2h at 37°C. The wells were washed three times with distilled water. Mice sera and BAL samples were added in 2-fold serial dilutions and plates incubated at 4°C overnight. The plates were then washed three times before the addition of goat anti-mouse immunoglobulin G (IgG) (Sigma, A2554), IgG1 (Southern Biotech, 1071-05), IgG2a (BD Pharmingen, 553391), IgA–horseradish peroxidase (HRP)-conjugated antibodies (Invitrogen, 62-6720) diluted 1:2000 in blocking buffer, except for the IgG2a antibody that was diluted 1:5000, at RT for 2h. Afterwards, plates were washed five times and the o-phenylenediamine dihydrochloride (OPD; SigmaFast OPD Peroxidase substrate. PCode 1003344899. Source SLCJ3691) substrate was added and incubated for 30 minutes at room temperature and the absorbance measured at 450nm in an ELISA plate reader (TECAN Sunrise). The data were expressed as endpoint titres calculated as double the background absorbance value.

### IgG and IgM detection in sera from TB patients

For the detection of PE/PPE-specific IgG and IgM in human sera, a similar protocol that was used for the serology studies in mice was applied. Following coating and blocking, the plates were incubated with anti-human IgG–HRP (Jackson 109:053-008) or anti-human IgM-HRP (Abcam ab97205) diluted 1:2000 in assay diluent for 2h at room temperature. OPD Substrate Tablets (Sigma-Aldrich) were used for colour development, following the manufacturer’s instructions. The OD at 450nm was measured after 15 minutes incubation at room temperature.

### PBMC re-stimulation assay in a Mozambican cohort

Cryopreserved PBMC were revived in R10 with 150µg/mL DNase I (Roche, 10104159001). After two wash steps with R10+DNase, cell viability was determined using Trypan Blue exclusion method. Only samples with a cell viability of more than 70% were used for the assay. Cells (5x10^5^ viable cells/well) were plated in U-bottom 96-well plates for each donor, having designated wells for unstimulated (media alone), positive control (PMA/ionomycin) and treatments (PE18, PE31 and PPE26). Antigen treated cells were stimulated with 5µg/mL of the respective antigen (PE18, PE31 or PPE26) for 18-24h. Six hours before cell harvest, positive control wells were stimulated with 10ng/mL PMA (Sigma, P1585) and 250ng/mL ionomycin (Sigma, I9657). Finally, all wells were treated with Brefeldin A (BioLegend, San Diego, CA, USA, 420601) (5µg/mL) 4h before FACS staining. Cells were washed with DPBS and stained with a master mix of 1:250 anti-human Fc receptor blocking antibodies (BioLegend, San Diego, CA, USA, 422302), 1:500 fixable viability dye eFluor 780TM (Invitrogen, 65-0865-14), 1:200 Brilliant Violet 421-conjugated anti-human CD3 (317344), 1:200 PerCP/Cy5.5-conjugated anti-human CD4 (357414) and 1:200 Brilliant Violet 510-conjugated anti-human CD8 (301048) (all from BioLegend, San Diego, CA, USA) for 45 minutes at 4°C. Cells were fixed and permeabilized for 15 minutes using IC Fixation buffer (Invitrogen, 00-822-49). Intracellular cytokine staining with 1:200 Alexa Fluor 700-conjugated anti-human IFN-γ (BD Biosciences, 557995), and 1:200 PE-Cyanine7-conjugated anti-human TNF (BioLegend, San Diego, CA, USA, 502930) was done with eBioscienceTM permeabilization buffer (Invitrogen, 00-8333-56). Stained samples were analysed within 24h after intracellular staining. Cell acquisition was performed using CytoFLEX S flow cytometer (Beckman-Coulter) and analysed using FlowJoTM v10.8.1 (BD Biosciences, Ashland, OR, USA). The frequencies of IFNγ+, TNF+ and IFNγ+TNF+ cells in total T cells, CD4 and CD8 T cells, were summed up and expressed as percentage of cytokine-positive cells. Gating strategy is shown in **Supplementary Figure 4**. Baseline values from unstimulated wells were deducted from stimulated wells to obtained proportion of stimulated cells.

### Preparation of BCG lysates

*Mycobacterium bovis* BCG (strain Pasteur) was cultured in Middlebrook 7H9 broth supplemented with 10% OADC enrichment for three weeks. The bacterial cells were harvested, washed, and lysed using a sonication method (UP200ST ultrasonic processor, Hielscher), by five 30s pulses with intermittent cooling. The lysate was centrifuged, and the supernatant containing the soluble BCG proteins was filtered using a 22µm syringe driven filter unit (Millex) and collected. Protein concentration was determined by measuring absorbance at 260/280 using nanodrop (Thermo Scientific, Nanodrop 2000). BCG lysate stocks were prepared at 1mg/mL concentration and frozen until further use.

### Western blotting for detection of antigen-specific antibodies against BCG

To determine the presence of PE/PPE antigens in the BCG Pasteur 1173P2 strain employed in this study, PE/PPEs (3µg) and BCG lysate (20µg) protein samples were separated by SDS-PAGE using 4-12% Bis-Tris polyacrylamide gels (Invitrogen) in MES running buffer (Invitrogen) for 1h at 100V 49mA. PE/PPEs were prepared in reducing and non-reducing conditions by addition of β-mercaptoethanol (Sigma) as reducing agent. The separated proteins were transferred onto PVDF membranes using a semi-dry transfer system. The membranes were blocked with 5% non-fat dry milk in Tris-buffered saline with Tween 20 (TBST) and then incubated with correspondent serum samples from immunized mice (1:500 dilution) overnight at 4°C. After washing with TBST the next day, the membranes were incubated with HRP-conjugated anti-mouse IgG secondary antibody (1:2000; Sigma). Protein-antibody complexes were visualized using an enhanced chemiluminescence detection system (Amersham ECL Prime).

### Detection of BCG antigen-specific antibodies by ELISA

High-binding ELISA plates (Nunc) were coated with respective antigens or BCG (6 or 20µg/mL) diluted in carbonate buffer and incubated as described earlier. After blocking the plates, diluted mouse serum samples (1:500 dilution) were added to the wells and incubated for 2h at room temperature. Following washing, HRP-conjugated anti-mouse IgG secondary antibody was added, and the plates were incubated for 2h. Substrate solution containing 3,3’,5,5’-tetramethylbenzidine (TMB) (Invitrogen) was added, and the reaction was stopped with 2N sulfuric acid after 25 minutes. Absorbance was measured at 450nm using a microplate reader.

### Generation of human DC

Peripheral blood mononuclear cells (PBMCs) from human healthy donors were thawed from ImmunXperts SA (Belgium) biobank. Monocytes were isolated from PBMCs using a MACS magnetic separation column CD14 MicroBeads (Miltenyi) and purity was evaluated by CD14 FACS staining (Fortessa). Cells were then resuspended at a cellular density of 10^6^ cells/mL and plated into a 24-well tissue culture microplate (1mL per well) in CellGenix DC medium (CellGenix, Cat.N° 20801-0500) added with Gentamycin, IL-4 (Miltenyi, 130-093-866) and GM-CSF (Miltenyi, 130-093-922) for 5 days. At day 5, cells were stained for FACS analysis with several DC activation markers to assess their immature dendritic cell (iDC) state: CD14-FITC (Miltenyi, 130-110-518), CD40-BV510 (BD Biosciences, 563456), CD80-BV421 (BD Biosciences, 564160), CD83-PE-Vio 770 (Miltenyi, 130-110-505), CD86-APC (Miltenyi, 130-116-161), CD209-PE (Miltenyi, 130-117-706), and HLA-DR-BUV395 (BD Biosciences, 564040). On the same day, respective antigens (10µg/mL) were added to the cell culture for 48h. At day 7, cells were stained for FACS analysis with same markers to assess their mature state. LPS (Sigma, L4391-1MG) (1µg/mL), and TNF (Miltenyi, 130-094-024) (800U/mL), with IL-1b (Miltenyi, 130-093-898) (150U/mL), were used as positive control.

### Human non-exposed PBMC proliferation assay

Human PBMCs from five healthy donors were thawed from ImmunXperts biobank and incubated with respective PE/PPE candidates (10µg/mL) for 7 days at 37°C. PBMCs from same donors were incubated with CEF (Mabtech, 3616-1) (HLA class I control) and CEFTA (Mabtech, 3617-1) (HLA class II control) peptide pools, a positive control for T cell activation. On day 6 of culture, the cells were incubated with 5-ethynyl-2’-deoxyuridine (EdU) (Thermofisher) (1μM) for 16h for assessment of T cell proliferation. The day after, supernatants from human PBMC stimulation assays were collected and stored at−80°C for further cytokine analysis. Each cell culture included a set of untreated control wells. Cells were stained for flow cytometry analysis of T cell surface markers (CD4 and CD8), fixed/permeabilized, and the incorporated EdU was stained with a fluorescent azide to assess T cell proliferation by measuring EdU uptake using flow cytometry.

### Human IFNγ ELISA

Cell supernatants were stored at −80°C and then analyzed for IFNγ expression with LEGEND MAX™ Human IFNγ Kit (BioLegend, San Diego, CA, USA) following the manufacturer’s instructions. Cytokine levels were determined by ELISA and absorption was read by using a microplate reader (Thermofisher). Each sample was tested in duplicate. The detection limit of the kit was 15.6-1000 pg/mL.

### Sample size and statistical analysis

Sample size for human cohorts was described above and was not based on power calculations but rather availability of samples from a previous exploratory study (EMI-TB project). Mouse groups for Mtb challenge experiment were based on power calculations and prior experience, where the minimal group size is six animals (seven were used, taking into account possible attrition). GraphPad Prism software, version V10 was used for determining the significant differences in the mean values between the samples when using parametric tests, or the mean rank of the data for non-parametric tests. For multiple group comparisons, one-way or two-way ANOVA tests with correspondent corrections were applied. In all, *p*-value of ≤0.05 was considered to be statistically significant.

## Results

### Antigen selection, expression and purification

**Figure 1:**
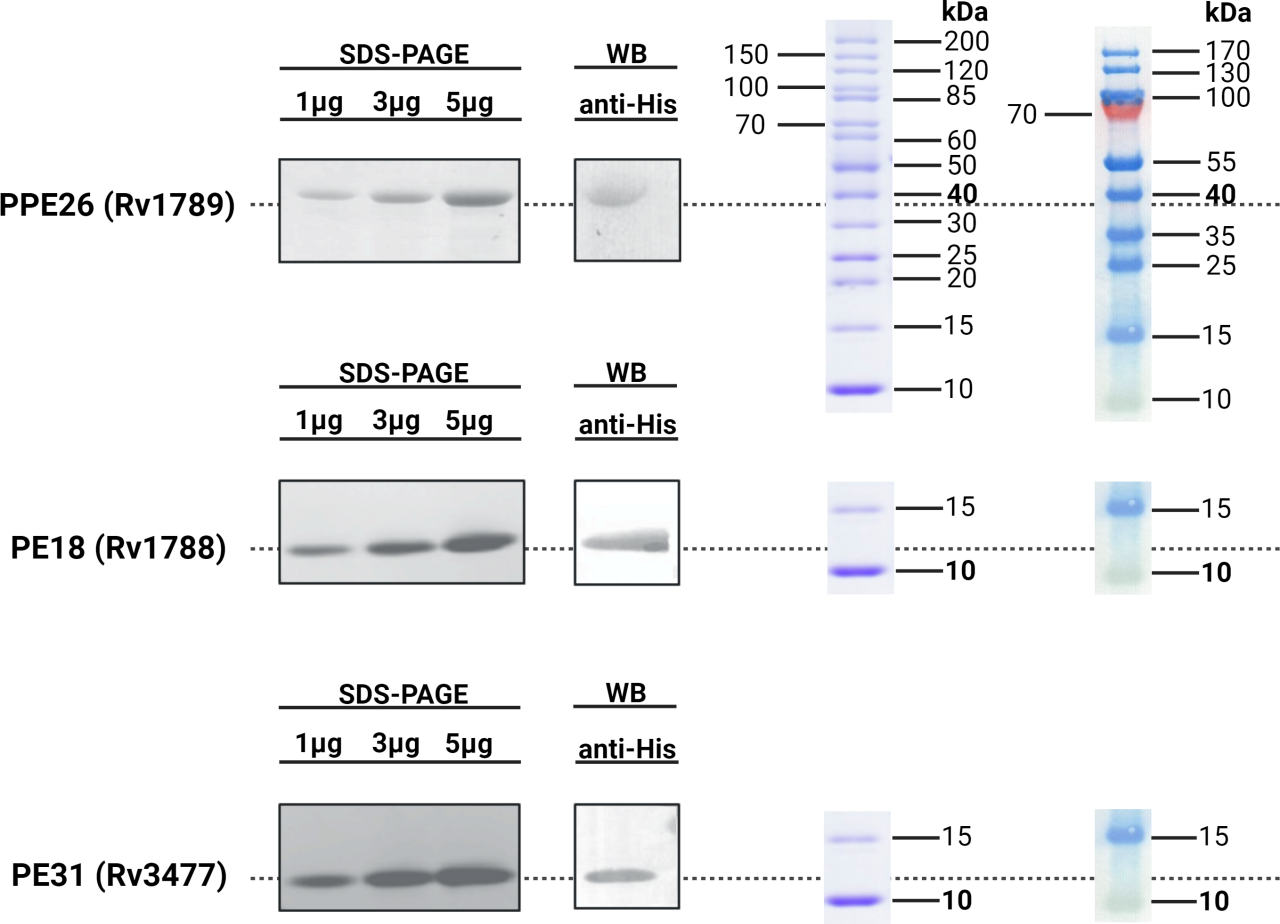
Immunoblot analysis of His-tagged recombinant Rv1789, Rv1788 and Rv3477. The purified recombinant proteins PPE26 (Rv1789), PE18 (Rv1788) and PE31 (Rv3477) of *Mycobacterium tuberculosis H37Rv* were separated by 15% SDS-PAGE and transferred to a PVDF membrane. These proteins were probed with polyclonal mouse anti-His antibody followed by goat anti-mouse IgG-HRP conjugated secondary antibody. Protein ladders for SDS-PAGE (left) and Western Blot (right) are included for molecular weight reference. All respective molecular weights with approximate expected sizes for proteins are highlighted in bold. Blots have been cropped for presentation purposes.

In order to investigate the immunogenicity and protective capacity of mycobacterial proteins we needed to generate quantities of pure protein. To do this, mycobacteria specific genes encoding proteins PE18, PE31 and PPE26 were expressed and purified as described before (28). Protein expression was induced with 1mM IPTG resulting in accumulation of insoluble proteins, and purity and precision of protein production confirmed by weight, with SDS-PAGE showing an apparent molecular weight of 9.8kDa for the PEs and 38kDa for PPE26. His-tagged protein products were isolated, affinity purified under denaturing conditions, and examined on 15% SDS-PAGE. Endotoxins were removed during the purification process (<10EU/mL). SDS-PAGE and immunoblot analysis were performed for the three proteins and are shown in **Figure 1**.

### PE18, PE31 and PPE26 are recognised by sera of TB, LTBI and HC (BCG) hosts

**Figure 2:**
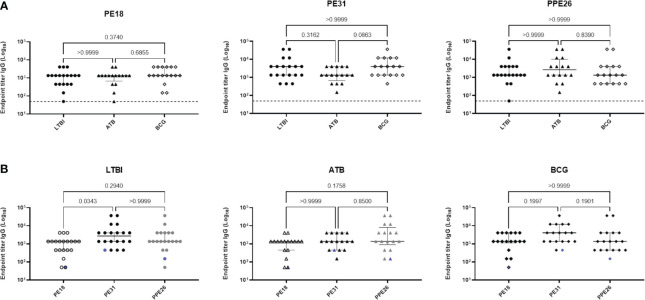
Human antibody response to Mtb proteins PE18, PE31 and PPE26. Serum derived from TB patients (n=16), IGRA-positive close contacts (n=19), and IGRA-negative BCG vaccinated healthy controls (n=17) from Mozambique were measured for antigen specific IgG antibodies **(A, B)**. The results are presented as individual values with median and interquartile range (IQR). For statistical analysis, non-parametric Kruskal-Wallis test was used. Multiple comparisons were corrected using the Dunn’s test. The significance levels are indicated numerically. The dotted line in **(A)** and the blue pattern symbols in **(B)** represent background antibody response from a European healthy donor with no history of TB or previous BCG vaccination.

To evaluate the immunogenic potential of PE18, PE31 and PPE26, we first analysed their immunoreactivity using sera derived from TB patients (n=16), IGRA^+^ close contacts (n=19), and IGRA^-^ BCG vaccinated healthy controls (n=17) from Mozambique (**Figure 2**). All three antigens were recognised by sera from TB-exposed individuals as well as LTBI hosts, as shown by antigen-specific IgG levels. The three proteins were also immunoreactive with sera of healthy BCG-vaccinated controls. There were no significant differences in their immunoreactivity between the three population groups, except for PE31, which showed a trend toward higher immunoreactivity with BCG-vaccinated healthy individuals compared to patients with TB, although this difference was not statistically significant (*p=0.0863*) (**Figure 2A**). Furthermore, as shown in **Figure 2B**, a comparison of the immunoreactivity levels of each of the three proteins per population group revealed that PE31 was recognized to a higher specific IgG antibody titre in latent TB patients compared to both PE18 and PPE26, with a significant difference found for PE18 (*p=0.0343*). As for the BCG-vaccinated control and the ATB groups, differences were also found with individuals exhibiting a higher PE31 titre, although this did not reach statistical significance. Notably, not all individuals were equally responsive. This may reflect not all subjects being diagnosed at the same stage of infection. In addition, we further measured the titres of antigen-specific IgM antibodies for a representative pool of five individuals per group (**Figure S3**). Sera from TB patients, IGRA^+^ household contacts, and IGRA^-^ BCG vaccinated healthy controls all reacted to all three proteins; however, PPE26 induced a weak antigen-specific IgM antibody response in IGRA^+^ household contacts (**Figures S3B**), while PE31 showed highest IgM antibody titres in this same group, resulting in significant differences (*p=0.0286*) when compared to PPE26 (**Figures S3-B**). PPE26 shows similar or higher proportion of antibody positivity in subjects from both the IGRA^-^ BCG vaccinated healthy control and the TB groups, but without being statistically significant. Importantly, no reactivity was detected from a European healthy donor with no history of TB or previous BCG vaccination. Overall, all three PE/PPE proteins are immunoreactive with sera from TB patients, IGRA^+^ household contacts, and also IGRA^-^ BCG vaccinated healthy controls from the Mozambique cohort.

### PE/PPE antigens are recognised by human PBMC and induce homeostatic-like T cell proliferation

**Figure 3:**
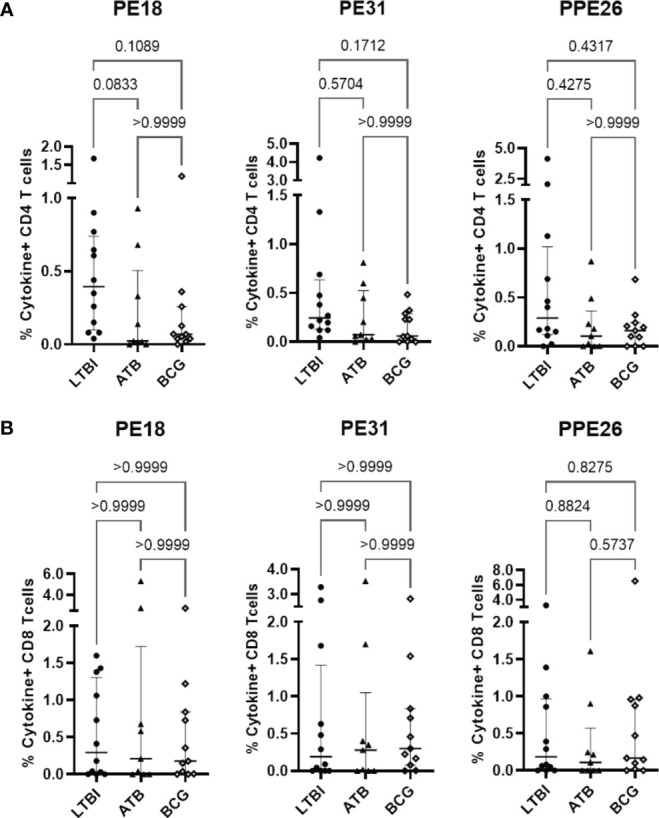
Antigen-specific T cell proliferation in PBMC from individuals exposed to Mtb or BCG. Human PBMC (n=12) from the Mozambique cohort that have been exposed to either Mtb (ATB or LTBI) or BCG were stimulated *in vitro* with each protein. For each antigen, the percentages of IFNγ and TNF positive antigen-specific CD4 **(A)** and CD8 **(B)** T cells in the LTBI, ATB, and BCG groups are depicted. The results are presented as median with IQR, showing the percentage of IFNγ+ TNF+ antigen-specific T cells. Statistical analysis was performed using the Kruskal-Wallis test followed by Dunn´s test correction.

While immunoglobulin responses indicate exposure to antigen, it is cellular responses that are considered critical for immunity to TB (31–33). To examine the potential for these antigens to induce cellular immunity, we measured proliferation and cytokine production in PBMC from individuals who have been exposed to either Mtb or BCG in response to our antigens of interest. Using a subset of participants from the Mozambique cohort, we observed Th1 (IFNγ and TNF) cytokine production by CD4+ T cells in LTBI, ATB, and BCG groups, with all three antigens (**Figure 3A**). We also observed a clear trend of highest production of these cytokines in PBMC from LTBI donors, though this did not reach statistical significance due to a small sample size (n=12). This trend was also observed when comparing all T cells producing IFNγ and TNF (**Figure S5**). In contrast, there were no differences in the CD8+ T cell subset, with all three cohort groups showing similar levels of cytokine producing cells (**Figure 3B**). When comparing the responses between the three PE/PPE antigens in each sub-cohort, again the highest frequencies were generally observed in LTBI individuals but there were no significant differences between the three antigens (**Figures S6-A, C**). This suggests that antigen-specific immune responses exist in all individuals exposed to mycobacteria, with LTBI hosts displaying a trend of higher antigen-specific CD4+ T cell frequencies than active patients or BCG-vaccinated controls.

The observation of broad stimulatory capacity of these antigens raised the question of how much of the response is antigen-specific T cell activation versus other non-specific stimuli. In a first analysis we measured the capacity of the proteins to alter the phenotype of human dendritic cells. **Supplementary Figure 7** indicates that none of the three PE/PPE proteins were able to alter surface expression levels of DC-associated activation markers (CD40, CD80, CD83, CD86), or of the pathogen recognition receptors (CD209/DC-SIGN) or antigen presentation (HLA-DR) molecules. We then tested the capacity of these proteins to induce proliferation of T cells from PBMC of healthy (but unscreened in terms of mycobacterial exposure) blood donors (**Figure S8**). Surprisingly, PE18 and PPE26, but not PE31, induced some CD4+ and CD8+ T cell proliferation in seven-day cultures, expressed as stimulation indices (**Figure S8**). Supporting these results, IFNγ was also detected in culture supernatants by ELISA. However, none of the three proteins showed significant differences relative to unstimulated control. While prior mycobacterial exposure (whether BCG or Mtb) could not be eliminated, it is also possible that some of the PE/PPE antigens may induce homeostatic-like proliferation of circulating memory T cells.

### Immunogenicity of proteins PE18, PE31 and PPE26 in C57BL/6 mice

**Figure 4:**
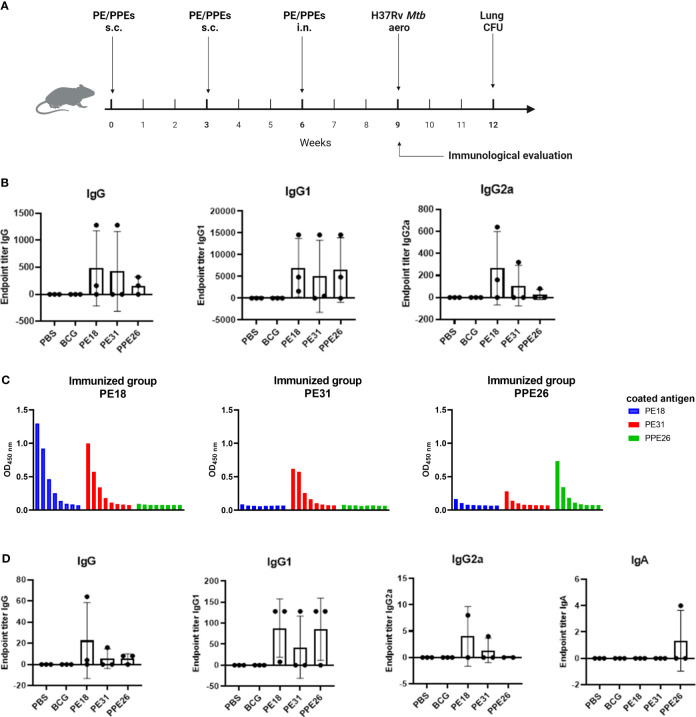
Antibody responses to Mtb recombinant antigens PE18, PE31 and PPE26 in immunized mice. **(A)** Mice were left unvaccinated (phosphate saline solution) or vaccinated either with BCG (5×10^5^ CFU BCG Pasteur) or 10µg of recombinant individual protein PE18, PE31 or PPE26 in combination with 1µg of adjuvant Quil-A, with immunisation schedule and subsequent Mtb challenge as schematically indicated. **(B)** Serum samples after the final immunization were subjected to ELISA to measure antigen-specific IgG, IgG1, and IgG2a antibodies. The endpoint titers were determined by calculating double the background absorbance value. Results show mean of reciprocal dilution ± SD. **(C)** PE/PPE proteins cross-react in serum from vaccinated animals. Serum samples from immunized mice were assessed for reactivity against the three proteins used in this study. The reactivity was determined using serial 3-fold dilutions ELISA and the absorbance at 450nm was measured. **(D)** Levels of antigen-specific IgG, IgG1 and IgG2a subtypes, as well as IgA antibody, were assessed in BAL from immunized mice similar to serum samples.

To investigate the T and B cell responses induced by the three vaccine candidates, C57BL/6 mice (n=3 per group) were immunized three times (two subcutaneous and one intranasal) 3 weeks apart with each individual antigen, BCG or PBS medium alone. Antigen-specific antibody titres and cytokine responses were determined three weeks after the last boost (**Figure 4A**).

Immunogenicity was assessed by measuring levels of antigen-specific IgG including individual subtypes IgG1 and IgG2a, in sera from immunized mice (**Figure 4B**). Mucosal immunogenicity was also analysed by levels of antigen-specific IgG and IgA in BAL samples (**Figure 4D**). The three PE/PPE proteins induced mixed IgG1/IgG2a antibody responses in sera as shown in **Figure 4B**. However, groups immunized with PE31 or PPE26 included some animals which appear to be non-responders for unknown reason, most probably due to suboptimal delivery. Furthermore, IgG1 titres in serum from immunized mice appear to be higher compared to IgG2a and total IgG, probably due to quality/sensitivity of secondary reagents (**Figure 4B**). Interestingly, we identified cross-reactivity within the sera against the three proteins (**Figure 4C**). Thus, the PE18-immunized mice not only responded with PE18-specific IgG but also showed cross-reactivity with PE31, probably due to the high level of homology between these two proteins (~64%). However, only PE31-specific IgG antibodies were identified in the PE31-immunized group with no cross-reactivity to either of the two other proteins. Nevertheless, two animals within the PE31-immunized group were poor responders, making definitive conclusions not possible. Mice immunized with PPE26 responded specifically to PPE26, with some weak cross-reactivity to PE31. Groups immunized with PBS or BCG that served as controls did not show any response to any of the three proteins, further suggesting that none of them is present in the BCG Pasteur 1173P2 strain used in this study (**Figures 4B, D**). To measure mucosal responses induced by the proteins, antibody levels were assessed in bronchoalveolar lavage (**Figure 4D**). BAL collected three weeks after final boost from animals immunized with PE18, PE31 or PPE26 showed relatively high levels of IgG as well as the IgG1 and IgG2a subtypes, especially for PE18. However, no antigen-specific IgA was detected in BAL (**Figure 4D**). Our results indicate that PE18, PE31 and PPE26 elicited humoral and cellular immune responses in humans and mice, which might contribute to protection.

### Absence of PPE26, PE18, and PE31 reactivity in BCG Pasteur suggests differential antigenic composition

To investigate the presence of PPE26 (Rv1789), PE18 (Rv1788) and PE31 (Rv3477) in the BCG Pasteur strain, mice were immunized with respective antigens, and their sera were collected for further analysis. ELISA and Western blotting were performed using the collected serum samples to evaluate reactivity of the three proteins to BCG (**Figure S9**). The findings demonstrated that immunized mice showed a strong immune response specifically directed at the respective antigens they were immunized with; however, no detectable reactivity was observed against BCG lysate in either of the mice that were immunized with the different antigens, as revealed by both ELISA (**Figures S9-A**) and Western blotting (**Figures S9-B**). Together, these results strongly suggest the absence of PPE26, PE18 and PE31 in the BCG Pasteur strain that we utilized in our study. However, other possibilities or alternative approaches like sequencing of the BCG Pasteur strain and potentially other BCG strains could provide definitive evidence whether the absence of these proteins is unique to the strain we used, or if it extends to other BCG strains as well.

### Polyfunctional T cells in splenocytes from immunized mice

**Figure 5:**
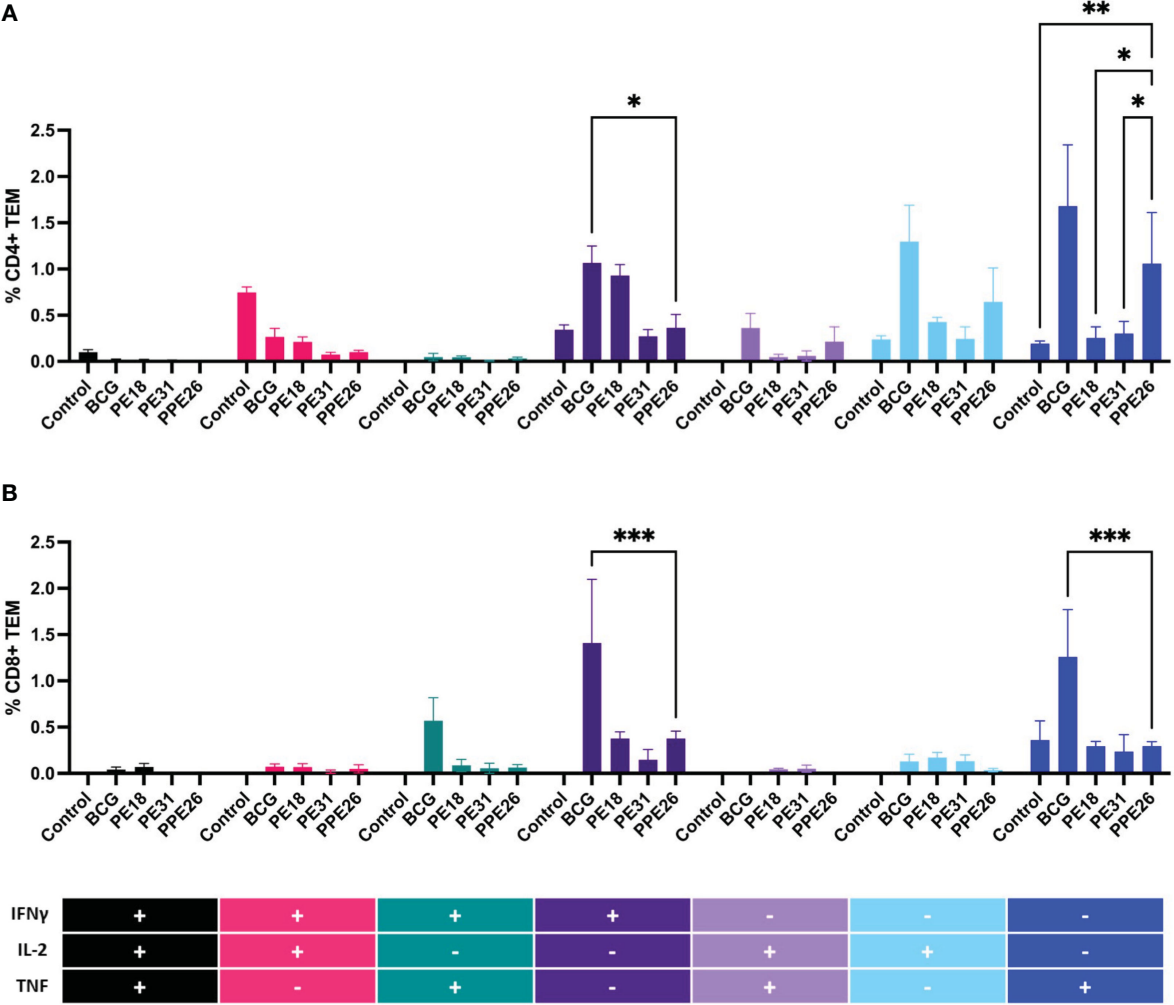
Antigen-specific CD4+ and CD8+ cell cytokine responses in vaccinated mice. Splenocytes from immunized mice were re-stimulated *in vitro* with respective proteins (10µg/mL) or BCG lysate, and stained for cell surface CD4 **(A)** and CD8 **(B)** molecules. Intracellular IFNγ, IL-2 and TNF were additionally stained. The percentage of cells expressing one cytokine or multiple cytokines IFNγ, TNF, and IL-2 was determined. The gating strategy for these experiments is shown in **Supplementary Figure S1**. The results represent mean values (n= 3 mice/group) for percentage of CD4+ or CD8+ T effector memory cells, with SEM. Asterisks denote significant differences according to a two-way ANOVA fit to a main effects only model. Dunnett test was applied for correction of multiple comparisons. The levels of significance are represented as follows: (*p < 0.05); (**p < 0.01); (***p < 0.001).

**Figure 6:**
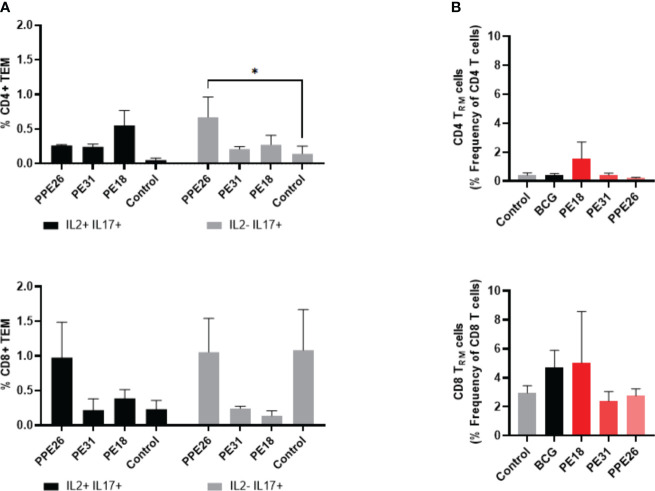
Antigen-specific CD4+ and CD8+ cytokine responses and tissue resident T cell populations in the lungs of immunized mice. **(A)** Antigen-specific CD4+ and CD8+ T cell cytokine responses in the spleens of immunized mice. The percentage of cells expressing IL-2, IL-17, or both cytokines was determined. The results represent mean values (n= 3 mice/group) with SEM for the percentage of CD4+ or CD8+ T effector memory cells, and were analyzed by a two-way ANOVA test followed by Dunnett correction test (*p < 0.05). **(B)** Total levels of CD4+ and CD8+ resident memory T cells were assessed in the lung tissue of vaccinated mice by flow cytometry, through staining of CD44+CD62L- and CD69+CD103+. The results show mean values (n= 3 mice/group) with SEM of percentage of CD4+ or CD8+ T resident memory cells. Statistical analysis was conducted using a one-way ANOVA test and a *post hoc* Tukey test.

**Figure 7:**
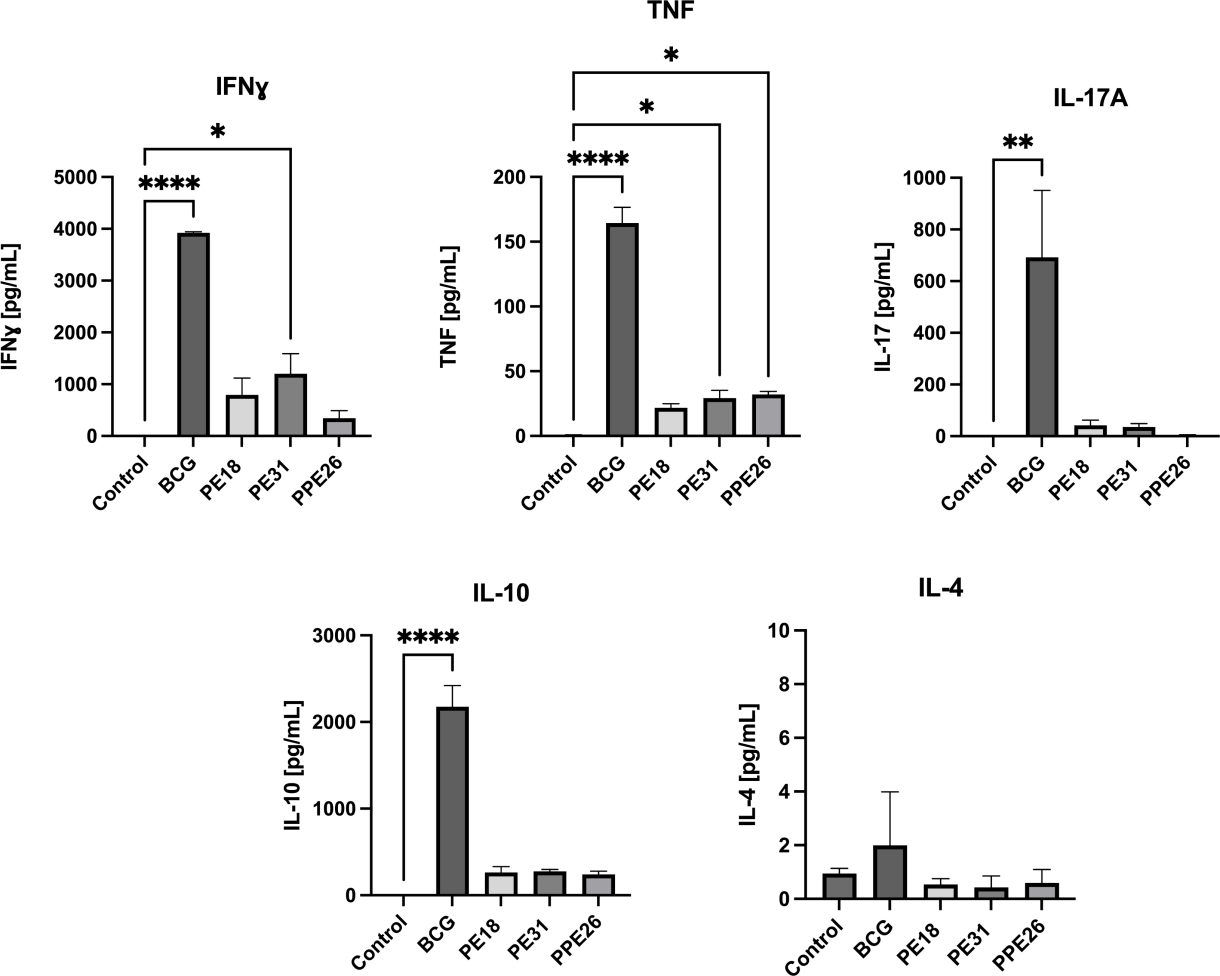
Cytokine production in splenocyte cultures supernatants. After re-stimulation with the respective antigens or with BCG lysate, cell culture supernatants were collected, and concentrations of IFNγ, TNF, IL-4, IL-10, and IL-17A were measured by quantitative ELISA. The data are presented as mean values with SEM. Statistical analysis was performed using one-way ANOVA followed by Dunnett test compared to control. (*p < 0.05, **p< 0.01, ****p < 0.0001).

To assess the ability of the vaccine candidate to induce specific functional T cells we examined the CD4+ and CD8+ T cell response to recall antigens. We used expression of multiple cytokines and effector molecules in response to stimulation with the specific antigens at the single cell level by intracellular cytokine staining (ICS) and flow cytometry. Expression of IFNγ, TNF and IL-2 (Panel A) or IL-2 and IL-17 (Panel B) in CD4+ and CD8+ T cells was determined (**Figures 5** **,** **6A**). Stimulation with PE18 as recall antigen led to an increased trend in the levels of IFNγ in both CD4+ and CD8+ T cell compartments in mice vaccinated with this specific antigen (**Figures 5A, B**), whereas in the case of PPE26-vaccinated mice, stimulation with PPE26 resulted in significantly higher levels of CD4+ T cells expressing TNF compared to the control group (**Figure 5A**). Additionally, there was an observed increased trend of CD8+ T cells expressing IFNγ alone in response to PPE26 (**Figure 5B**). The observed trends in increased levels of IFNγ and TNF in response to PE18 and PPE26, respectively, suggest a potential Th1 response in both instances. Furthermore, as shown in **Figure 6A**, splenocytes from PPE26 immunized animals resulted in a significant number of IL-17 cytokine producing CD4+ T cells (0.674%), suggesting a potential role for PPE26 in inducing a Th17 response. A similar trend was seen in CD8+ T cells, although it did not reach statistical significance, likely due to high variability in the saline control group (**Figure 6A**). Although there was an increase in the frequencies of CD4+ and CD8+ T cells showing IL-2+IL-17+ expression upon re-stimulation with each of the individual proteins as compared to the unstimulated control, these differences did not reach statistical significance (**Figure 6A**). We analyzed cytokine secretion in the supernatants from the re-stimulation assays using ELISA (**Figure 7**). Compared to the control, IFNγ levels were elevated for all three antigens, with significant differences found upon re-stimulation with PE31. Notably, significant differences in TNF secretion were detected for PE31 and PPE26 upon recall compared to the unstimulated control. Additionally, although not significant, elevated levels of IL-10 were observed in response to re-stimulation by all three antigens (**Figure 7**). Conversely, levels of IL-17A, although not statistically significant, were increased compared to the control, with PPE26 being the lowest inducer in comparison to the other two PE antigens. Finally, re-stimulation with each of the three antigens did not result in an increase of IL-4. Taken together, these results indicate that PE18, PE31 and PPE26 predominantly elicited Th1-type CD4+ and CD8+ effector T cell immune responses in C57BL/6 mice.

### PE18 stimulates resident memory T cells (Trm) in the lungs

We investigated if there was evidence of T cell resident memory (Trm) in the lungs of mice immunized with PE18, PE31 and PPE26, and used flow cytometry to quantify these cells in the lung homogenates. As seen in **Figure 6B**, the total numbers of Trm as defined by the CD69+ CD103+ phenotype for both the CD4+ and CD8+ compartments, were very low for BCG immunized animals, probably due to the vaccine having been given subcutaneously, while naïve animals showed only background levels (**Figure 6B**). In contrast, there was an increase in Trm populations in the PE18-immunized group for both T cell compartments, though this did not reach statistical significance, possibly due to large variations between the three animals. PE31 and PPE26 did not show the same trend of Trm increase, in either compartment. It should be noted though that our analysis was restricted to the total Trm population rather than antigen-specific Trm cells in the lungs.

Nevertheless, considering the experimental design where each group was immunized with a single antigen, and the mice were housed under controlled indoor conditions, it is reasonable to attribute any observed differences between groups to the respective vaccine candidates. While other possibilities exist, the consistent delivery of antigens suggests that intrinsic reasons for distinct mucosal responses are less likely. Finally, the fact that there was an increase in Trm populations within the PE18-immunized group for both CD4+ and CD8+ T cell compartments suggests that only immunization with PE18 might have triggered a localized immune response in the lungs.

### PE18, PE31 and PPE26 proteins did not confer protection against Mtb

**Figure 8:**
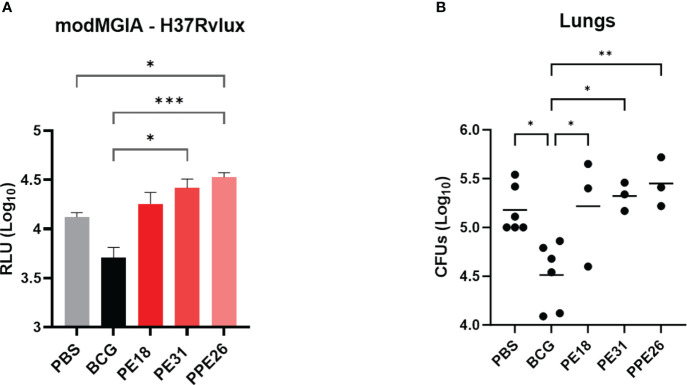
Assessment of protective efficacy by PE/PPE antigens in immunized mice. The figure depicts 2-fold evaluation of the protective response induced by PPE26, PE18 and PE31, as compared to negative and BCG control groups. **(A)** Splenocytes from immunized mice were evaluated for their ability to restrict mycobacterial growth *in vitro* using mycobacterial growth inhibition assay (MGIA). Splenocytes were subjected to a co-culture period with the Mtb-LUX strain, and luminescence measurements were employed as a surrogate for determining viable bacterial counts. Data are expressed as relative light units (RLU). **(B)** Lung homogenates were examined for colony-forming units (CFUs) after a 3–4-week incubation period. In both assays, one-way ANOVA with Tukey *post-hoc* test was performed. The data are presented as mean values with SEM. P-value of ≤0.05 was considered to be significant. (*p < 0.05, **p < 0.01, ***p < 0.001).

To address the capacity of the candidate proteins to induce protective effects, we first tested immunised mouse splenocytes for the ability to restrict mycobacterial growth *in vitro*, in a modified MGIA assay. Following 5 days co-culture with Mtb-LUX strain, we measured luminescence as a proxy for viable bacterial count. The results shown in **Figure 8A** indicate that splenocytes from BCG-immunised mice resulted in an approximate 0.5 log-fold reduction in bacterial count, compared to unimmunised mice. However, none of the PE/PPE proteins induced a significant effect on bacterial growth inhibition. To assess the ability of the vaccines to induce lung specific immunity we determined viable Mtb counts in lung homogenates from vaccinated mice, in a standard colony-forming unit (CFU) assay. The number of viable bacilli (CFU) in the lungs expressed as mean lung Log10 CFU was reduced from 5.2 in the unvaccinated group to 4.5 in BCG vaccinated mice. However, no reduction was observed as a result of vaccination with the PE/PPE proteins (**Figure 8B**), though unfortunately, due to technical issues, only three animals were assessed. Taken together, the MGIA assay and the bacterial plating suggest that none of the three PE/PPE proteins tested were protective in the mouse model of infection with Mtb.

## Discussion

In this study, we investigated the immunogenicity of PPE26, PE18 and PE31 proteins of Mtb H37Rv in both humans exposed to Mtb, and in mice vaccinated with the proteins.

In the quest for a successful TB vaccine, the research has been dedicated to identifying vaccine candidates that can trigger the generation of memory T cells exhibiting a Th1 phenotype. This involves the production of crucial cytokines such as IFNγ, TNF, and IL-2. These CD4+ Th1 lymphocytes and the IFNγ they produce are critical in controlling Mtb infection, as evidenced by susceptibility to mycobacterial disease in individuals with deficiencies affecting components of the IFNγ pathway (34). Notably, the secretion of IFNγ upon re-stimulation of cells exposed to the pathogen stands as a significant marker for cellular immune responses in Mtb infections (35). In the current investigation, we conducted *in vitro* re-stimulation assays to assess the proliferation and cytokine production, specifically IFNγ and TNF, by CD4+ and CD8+ T cells in response to PE18, PE31 and PPE26 proteins. These assays were performed using PBMCs from a cohort from the TB endemic region of Mozambique, which consisted of individuals with exposure to Mtb (both ATB and LTBI) as well as individuals who had received BCG vaccination (BCG-vaccinated healthy controls). Our results showed that the three antigens elicited Th1 cytokine (IFNγ+TNF+) production by CD4+ and CD8+ T cell subsets in individuals from the ATB, LTBI, and BCG-vaccinated groups. Notably, a trend towards higher cytokine production was observed in LTBI individuals, indicating the potential importance of these antigens in the context of latent infection. Although this trend did not reach statistical significance, it is consistent with previous findings indicating differential T cell responses in subjects with latent infection and active disease (36, 37). Latency-associated antigens, such as Rv1733c, have been reported to lead to higher numbers of T cells producing elevated levels of IL-2 in LTBI compared to ATB, supporting their potential for differential diagnosis (38). This suggests a potential association between the nature of the three PE/PPE proteins and latency. In our study, the lack of significant differences among the three groups may be influenced by various factors, including the prolonged exposure of LTBI individuals to the TB pathogen, potential immune compromise in active TB patients, and the specificity of T cell responses in BCG-vaccinated individuals. These findings indicate that the immune response to these proteins is not limited to active TB disease, but can be elicited in various TB or other mycobacterial exposure contexts. Additionally, the presence of antigen-specific antibodies against all three antigens in the sera of individuals across all three groups further supports these results. Importantly, we observed that not all individuals within the groups exhibited equal responsiveness, which could potentially be attributed to variations in the stage of infection at the time of diagnosis. Given the observation of broad T cell stimulatory capacity of these antigens, we also tested if the three antigens might have immunomodulatory properties in PBMC from non-exposed individuals. It has been shown previously that naive T lymphocytes can undergo heterogeneous proliferative responses (homeostatic proliferation) (39). Homeostatic proliferation, driven primarily by cytokines such as IL-7 and IL-15 rather than specific antigen recognition (40), is a gradual response. This slow nature could explain the observed lower levels of CD4 and CD8 T cells in PBMCs from non-exposed individuals in our study. This process needs of TCR interaction with MHC:peptide/antigen complexes and signals from cytokine receptors for T cell proliferation (41). Our findings suggest that these antigens may trigger a mechanism comparable to homeostatic T cell proliferation in PBMCs from individuals without prior exposure to Mtb or BCG. This response was observed upon stimulation with PE18 and PPE26, but not PE31. The observed proliferation of CD4+ and CD8+ T cells, along with increased levels of IFNγ upon stimulation with these proteins, suggest either some form of antigen cross-reactivity (such as environmental mycobacteria), or it could mean that they possess some inherent homeostatic properties, driving expansion of the general T cell memory populations in the absence of antigen presentation, a phenomenon that requires further investigation.

In this study, to delve deeper into the T and B cell responses prompted by the three vaccine candidates, C57BL/6 mice were immunized with the respective proteins and their potential to induce both humoral and cellular immune responses was evaluated. The choice of immunization routes, including two subcutaneous and one intranasal, was strategically planned to harness dual benefits. While intranasal immunization provides a localized defense at the site of initial infection (33, 42), subcutaneous immunization elicits a robust systemic immune response, capable of controlling the spread of the pathogen to other organs. First, we analysed reactivity to PE/PPE antigens in serum and BAL of immunized animals. Measured levels of antigen-specific IgG and IgA antibodies indicated a systemic rather than mucosal immune response. Furthermore, PE18 cross-reacts with PE31, and PPE26 has some degree of cross-reactivity with PE31, as showed by levels of antigen-specific IgG. The differential cross-reactivity observed between different PE/PPE proteins could be due to variations in antigenic similarity, with PE18 and PE31 sharing relatively high homology (~64%). Such cross-reactivity could be harnessed in vaccine development, as targeting multiple antigens with cross-reactive epitopes may enhance the breadth and potency of the immune response. Unlike our findings in humans, which demonstrated immunogenicity of PE/PPE antigens in individuals exposed to Mtb and BCG vaccination, mice immunized with BCG exhibited no response to any of the three proteins. This observation suggests that these antigens may be absent in the BCG Pasteur 1173P2 strain utilized in this investigation. To further confirm those results, we performed WB and ELISA analyses with sera from mice immunized with the respective antigens, and assessed their reactivity against the BCG Pasteur strain used in this study. Again, the absence of reactivity in BCG by either WB or ELISA conclusively indicates that the three proteins are not present or expressed in significant quantities in the BCG Pasteur strain. These contrasting observations in humans and mice raise the intriguing question about the nature of the immune response elicited to these antigens in the BCG cohort from Mozambique. The most likely explanation is the interference from NTM, which share numerous antigens with Mtb and BCG, including proteins from the distinctive PE/PPE family. Furthermore, the exact composition of PE/PPE proteins in BCG varies depending on the strain. As an example, Abdallah et al. found over-expressed levels of ESX5 locus genes *pe18* and *ppe26* exclusively in BCG Tice as a result of ESX5 locus duplication (24). Therefore, it is possible that the immune response observed in BCG-vaccinated individuals is a result of cross-reactivity with shared antigens or epitopes present in some NTM bacteria, rather than direct recognition of the proteins in the BCG vaccine strain. Nevertheless, further investigations are warranted to elucidate the specific antigenic components of BCG and their interactions with the host immune system, ultimately enhancing our understanding of BCG-mediated immunity and its potential for improved vaccine development strategies. Further, these findings raise important questions about the potential benefits of incorporating these antigens into BCG-based vaccine strategies. Based on these insights, the concept of using a BCG prime/protein boost strategy with these antigens becomes even more significant. This notion implies that incorporating these antigens into the vaccination scheme could address potential limitations in BCG’s efficacy and amplify the immune reaction against tuberculosis.

We also evaluated T cellular proliferation and cytokine responses in immunized mice. In addition to antibody production, our results showed that the three proteins, especially PE18 and PPE26, were capable of inducing activation of both CD4+ and CD8+ T cell populations, accompanied by the secretion of Th1-associated cytokines such as IFNγ and TNF, upon re-stimulation with specific antigens. Furthermore, augmented levels of IL-2 and IL-17 were observed upon re-stimulation with all three proteins, in both the CD4+ and CD8+ compartments. Likewise, the increase in IFNγ and TNF secretion by all three antigens indicates their potential to elicit pro-inflammatory responses, which however appear self-limiting, due to also elevated trends of the regulatory cytokine IL-10. Additionally, in this study, none of the antigens led to the release of IL-4, as measured in the supernatants during antigen re-stimulation assays. This reinforces the idea that the immune response triggered by these antigens is favouring a Th1 profile rather than a Th2 response. In our study, we also observed a noticeable trend towards an increase in lung resident memory T cells among mice vaccinated with PE18, although the specificity to the antigen was not confirmed. The lack of a similar response in the other two groups could indicate antigen-specific variations in immune interactions. While the study’s design has limitations, the controlled environment and consistent antigen delivery lend credibility to the idea that the observed differences are likely attributed to the antigenic stimuli themselves. Thus, the trend observed in the PE18-immunized group might indicate the potential presence of a mucosal immune response in the lungs of animals immunized with this specific antigen. Further investigation, including antigen-specific Trm analysis, could provide more precise insights into the impact of these antigens on lung-resident immunity.

Despite these promising immune characteristics, none of the tested PE/PPE proteins conferred protection in the mouse model of TB, as evidenced by the lack of reduction in the lung CFU counts. These findings suggest that while the PE/PPE proteins are capable of inducing broad immune responses, including antibody production and T cell memory, this does not translate into protective immunity against TB, at least in the mouse model of infection. However, several factors may contribute to this outcome. Firstly, the approach of using subunit vaccines, as employed in our study with individual proteins and the Quil-A adjuvant, may not fully capture potential synergistic effects achievable by combining multiple proteins or even PE/PPE complexes. Additionally, the timing of infection and the assessment of immune responses can significantly impact the observed results. In our study, bacterial burdens in the lungs were assessed three weeks after Mtb challenge, a relatively short period. Contrasting studies (16) conducted assessments one to three months following aerosol infection, suggesting that longer evaluation periods might better capture protective effects or potential changes in immune responses over time. Furthermore, considering the observed levels of CFU counts and results from the MGIA assay, which indicated higher trends of bacterial loads in the PE/PPE-vaccinated animals compared to controls, particularly for antigen PPE26, raises the possibility that these proteins might not confer protection, but instead, could enhance bacterial burden. In this context, several Mtb proteins have been reported to evoke innate and adaptive immune responses, though many of these act as decoy antigens, mimicking host-pathogen effector components and misdirecting immune response pathways to favour the pathogen’s survival (43, 44). Thus, PPE26 antigen may play a role in the bacterium’s activation of immune evasion mechanisms or modulation of immune responses, interfering with the host’s immune defence mechanisms and potentially creating a more favourable environment for bacterial growth. Future research could delve into the mechanisms by which these proteins may affect disease progression and explore modifications or combinations to mitigate any unintended consequences. In this regard, the combination of multiple antigens can elicit a more robust immune response compared to the individual proteins alone. This was shown, for example, when vaccination with adjuvanted individual Rv1789, Rv2220, or Rv3478 proteins conferred only partial protection against challenge with Mtb, but improved protection when the three were administered together (16). Similarly, Bertholet et al. investigated the protective efficacy of a combination of antigens (Rv2608, Rv1813, and Rv3620) against Mtb infection. Individually, these antigens provided partial protection; however, when administered as a combination, a marked increase in protection was observed, comparable to that achieved with BCG vaccination (15). These findings emphasize the potential benefits of combining multiple antigens to enhance the immune response by increasing the production of antigen-specific antibodies, activation of T cells, and release of cytokines involved in immune regulation and pathogen clearance. Furthermore, PE/PPE complexes are of particular interest due to their unique immunomodulatory properties (45). While individual antigens like PE35 and PPE68 are immunogenic individually, their corresponding complex, PE35/PPE68, exhibited significantly higher antibody response in mice (46). Similarly, the PE25/PPE41 complex elicits stronger immune responses compared to individually expressed PE25 or PPE41 proteins in TB patients as well as in a TB mouse model (47). It could therefore be argued that PE18 and PPE26 antigens that can form a potential PE/PPE complex (28, 48), could be more immunogenic and protective if used together, as opposed individually, as performed in this study. Moreover, the possibility of improving overall protection and expanding immune coverage by combining PE/PPE-based vaccines with established TB vaccines like BCG offers an interesting path for further investigation. Finally, additional factors beyond Th1 responses (32) might be important in controlling the Mtb infection in these mice. These results are consistent with previous findings from other studies and underscore the complex and diverse nature of immune responses in the fight against tuberculosis (49, 50).

In conclusion, our study highlights the immunogenicity of PPE26, PE18 and PE31 from Mtb and their ability to elicit broad antibody and T cell responses. However, the lack of protective efficacy observed underscores the need for a comprehensive understanding of the underlying mechanisms and potential synergistic interactions among different PE/PPE proteins. Further research in this area could lead to the development of new TB vaccine strategies that incorporate members of the PE/PPE protein family into their formulation.

## Data availability statement

The original contributions presented in the study are included in the article/**Supplementary Material**. Further inquiries can be directed to the corresponding author.

## Ethics statement

The studies involving humans were approved by the Mozambican National Bioethics committee (IRB:00002657; ID: 298/CNBS/15). The studies were conducted in accordance with the local legislation and institutional requirements. The participants provided their written informed consent to participate in this study. The animal study was approved by the University of Leicester Ethics Committee under an approved UK Home Office animal project license (Establishment License X1798C4D2) and in accordance with the Animals (Scientific Procedures) Act 1986. The study was conducted in accordance with the local legislation and institutional requirements.

## Author contributions

MG-B: Conceptualization, Data curation, Formal analysis, Funding acquisition, Investigation, Methodology, Project administration, Resources, Software, Visualization, Writing – original draft, Writing – review & editing. EV: Conceptualization, Data curation, Formal analysis, Funding acquisition, Investigation, Methodology, Project administration, Resources, Software, Visualization, Writing – review & editing. AT: Conceptualization, Data curation, Formal analysis, Funding acquisition, Investigation, Methodology, Project administration, Resources, Software, Visualization, Writing – review & editing. LB: Data curation, Formal analysis, Methodology, Software, Writing – review & editing. AC: Conceptualization, Data curation, Formal analysis, Investigation, Methodology, Validation, Visualization, Writing – review & editing. JP: Conceptualization, Data curation, Formal analysis, Investigation, Methodology, Validation, Visualization, Writing – review & editing. TM: Resources, Writing – review & editing. Mv: Formal analysis, Supervision, Writing – review & editing. MS: Funding acquisition, Project administration, Resources, Supervision, Writing – review & editing. RR: Conceptualization, Formal analysis, Funding acquisition, Project administration, Resources, Supervision, Writing – review & editing.
