# Supplementary material for: Immunogenicity of PE18, PE31, and PPE26 proteins from Mycobacterium tuberculosis in humans and mice
Source: Front Immunol. 2023 Dec 6;14:1307429. doi: 10.3389/fimmu.2023.1307429 (PMC10730732; doi:10.3389/fimmu.2023.1307429)
Supplement: Supplementary file 1 [file DataSheet_1.docx]

**Immunogenicity of PE18, PE31, and PPE26 proteins from *Mycobacterium tuberculosis* in humans and mice**

**María García-Bengoa^1,2,3*^, Emil Joseph Vergara^4*^, Andy C. Tran^4*^, Lorenzo Bossi^5^, Andrea M. Cooper^6^, John E. Pearl^6^, Tufária Mussá^7^, Maren von Köckritz-Blickwede^1,2^, Mahavir Singh^3^, Rajko Reljic^4^**

^1^Institute of Biochemistry, University of Veterinary Medicine Hannover, Germany

^2^Research Center for Emerging Infections and Zoonosis (RIZ), University of Veterinary Medicine Hannover, Germany

^3^LIONEX Diagnostics and Therapeutics GmbH, Braunschweig, Germany

^4^Institute for Infection and Immunity, St. George’s University of London, London, United Kingdom

^5^Immunxperts SA, a Q² Solutions Company, Gosselies, Belgium

^6^Department of Infection, Immunity and Inflammation, University of Leicester, UK

^7^Eduardo Mondlane University, Mozambique

*These authors contributed equally to this work.

§ **Correspondence:** María García-Bengoa Maria.Garcia.Bengoa@tiho-hannover.de

# Supplementary Figures and Tables

## Supplementary Figures

**
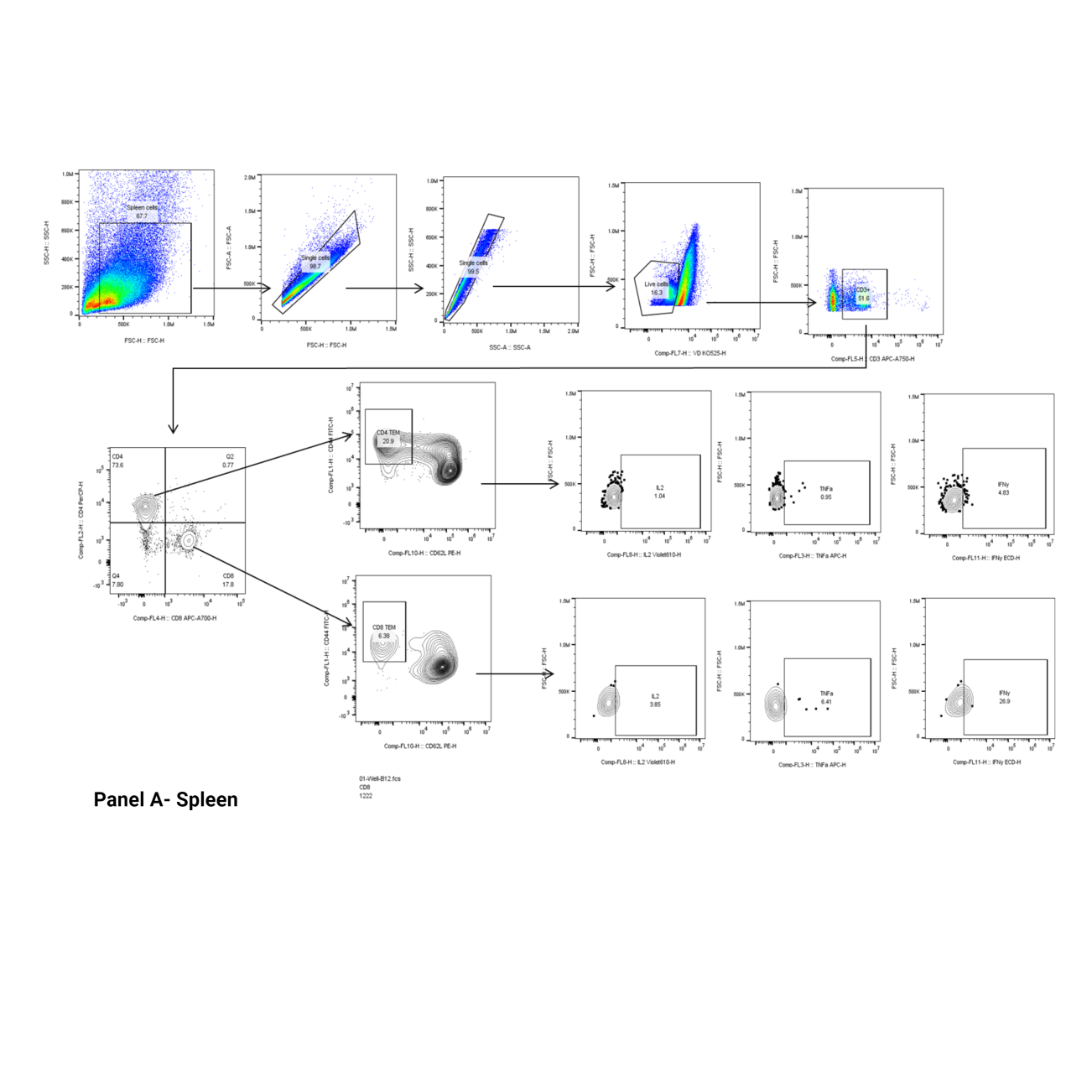
**

**Supplementary Figure 1.** Sequential gating for flow cytometry analysis of spleen cells from immunized mice. Initial gating was performed based on forward scatter area (FSC-A) versus forward scatter height (FSC-H) parameters to select single cells for subsequent analysis. To exclude non-viable cells, cells were gated based on viability dye fluorescence. Then, resultant live cells were further analyzed to identify CD3+ T cells. Within the CD3+ cell population, CD4+ and CD8+ T cells were identified with specific markers and resultant populations were further analyzed based on CD44+CD62L- to distinguish activated effector memory (Tem) CD4+ or CD8+ T cells, respectively. Within the resultant CD4+ T cell and CD8+ T cell Tem populations, the expression of specific cytokines like IL-2, TNF, or IFNγ (Panel A) was assessed.


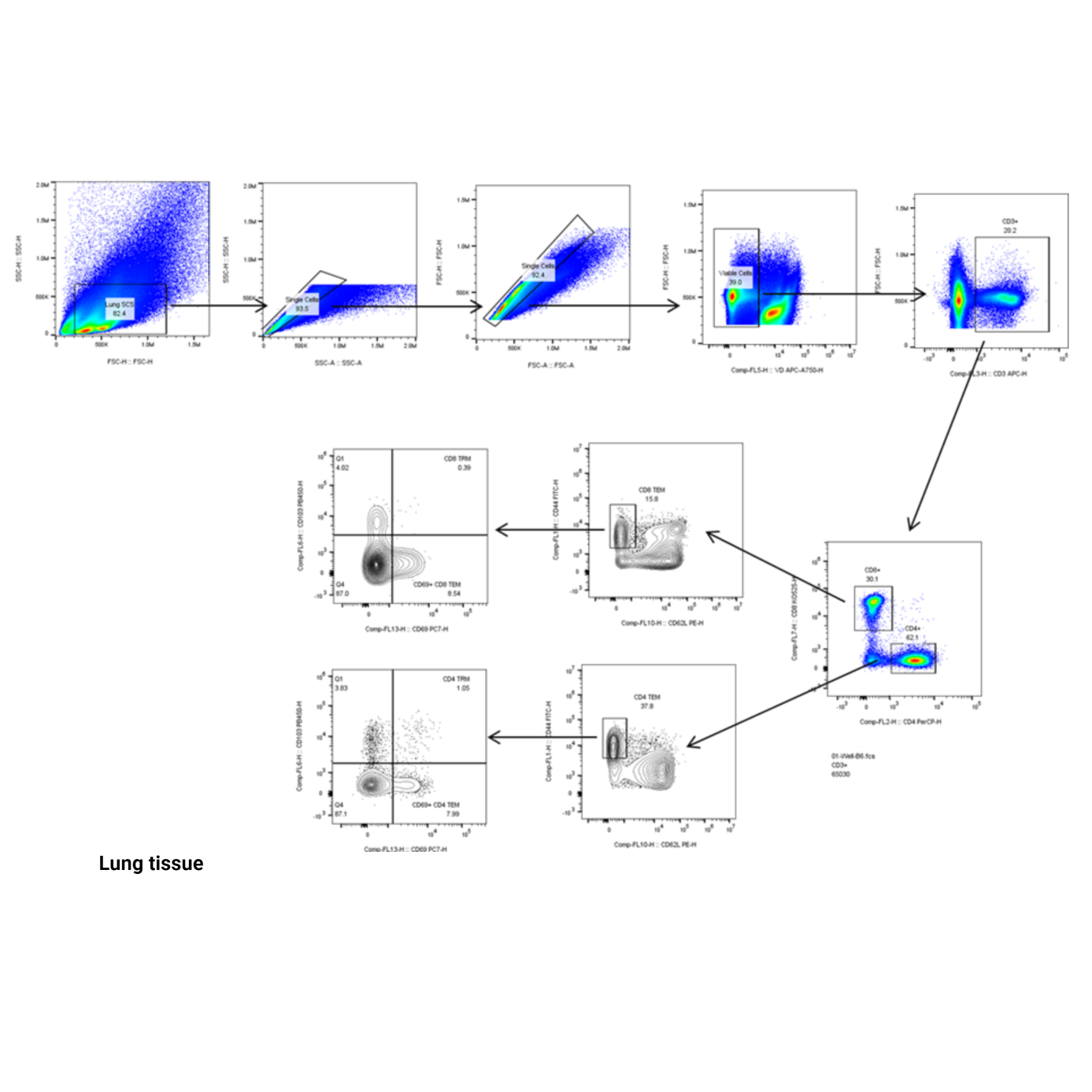


**Supplementary Figure 2**. Sequential gating strategy for flow cytometry analysis of lung resident memory cells from vaccinated mice. The initial gating process involved the discrimination of single cells based on forward scatter area (FSC-A) versus forward scatter height (FSC-H) parameters. Viability dye fluorescence was utilized to exclude non-viable cells from the analysis. The resulting live cells were subjected to identification of CD3+ T cells. Within the CD3+ cell population, specific markers were employed to distinguish CD4+ and CD8+ T cells. The subsequent analysis involved the characterization of activated effector memory (Tem) CD4+ and CD8+ T cells based on CD44+ and CD62L-. In the CD4+ and CD8+ Tem cell populations, the expression of the T resident memory (Trm) cell markers CD69 and CD103 was evaluated.


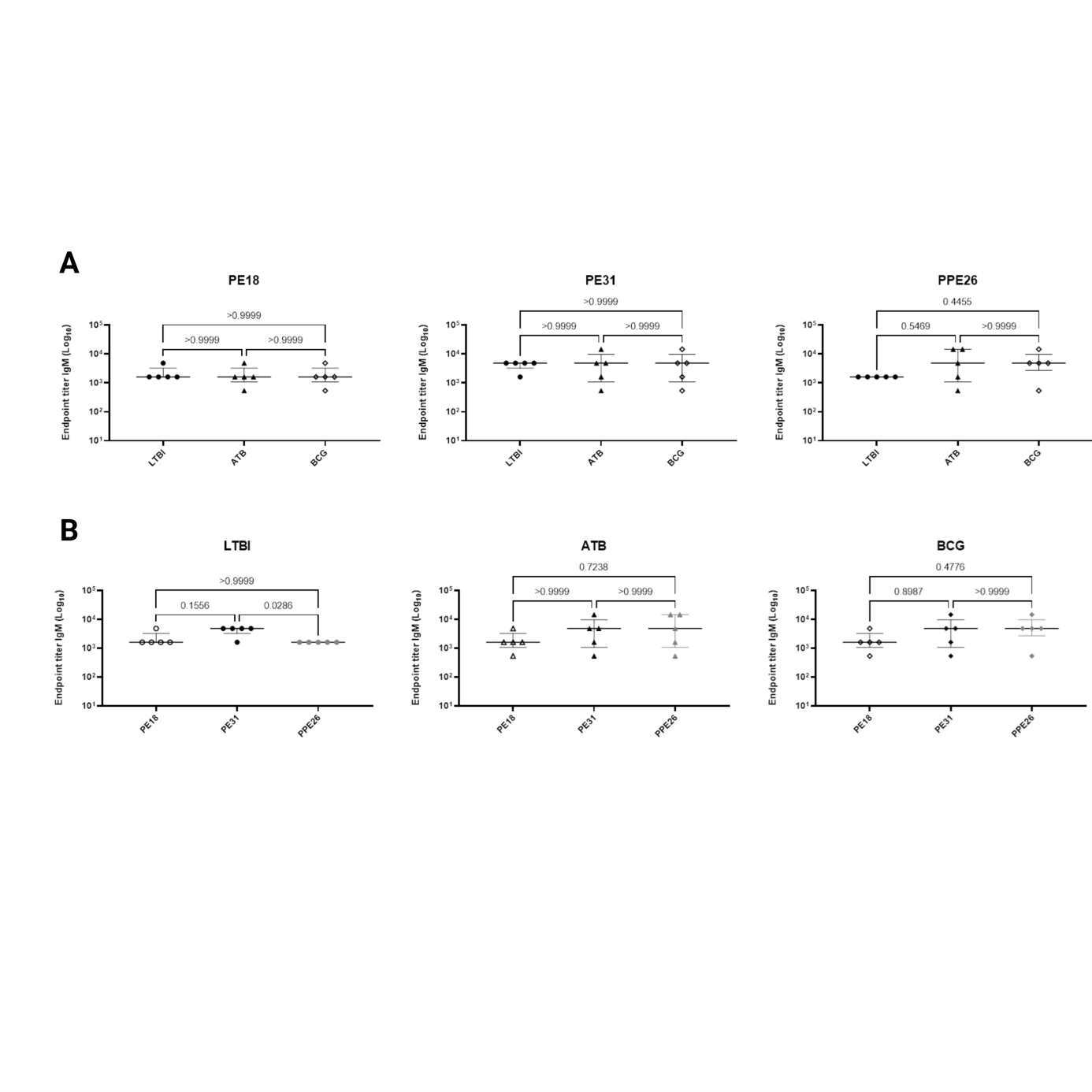


**Supplementary Figure 3.** Human antibody response to *Mtb* proteins PE18, PE31 and PPE26 in different TB populations. Serum samples were collected from individuals with active TB, LTB infected individuals, and IGRA-negative BCG vaccinated healthy controls from Mozambique. For this experiment, each population group consisted of five individuals, providing robust representation within each group. A healthy donor with no previous history of BCG vaccination served as control (represented cero). The levels of antigen-specific IgM antibody **(A-B)** were measured using quantitative ELISA assay. These results represent individual values by the median and the interquartile range (IQR). Statistical analysis were performed using a Kruskal-Wallis test for non-parametric analysis. Multiple comparisons were adjusted using the Dunn´s test for correction. The levels of significance are indicated as numbers.


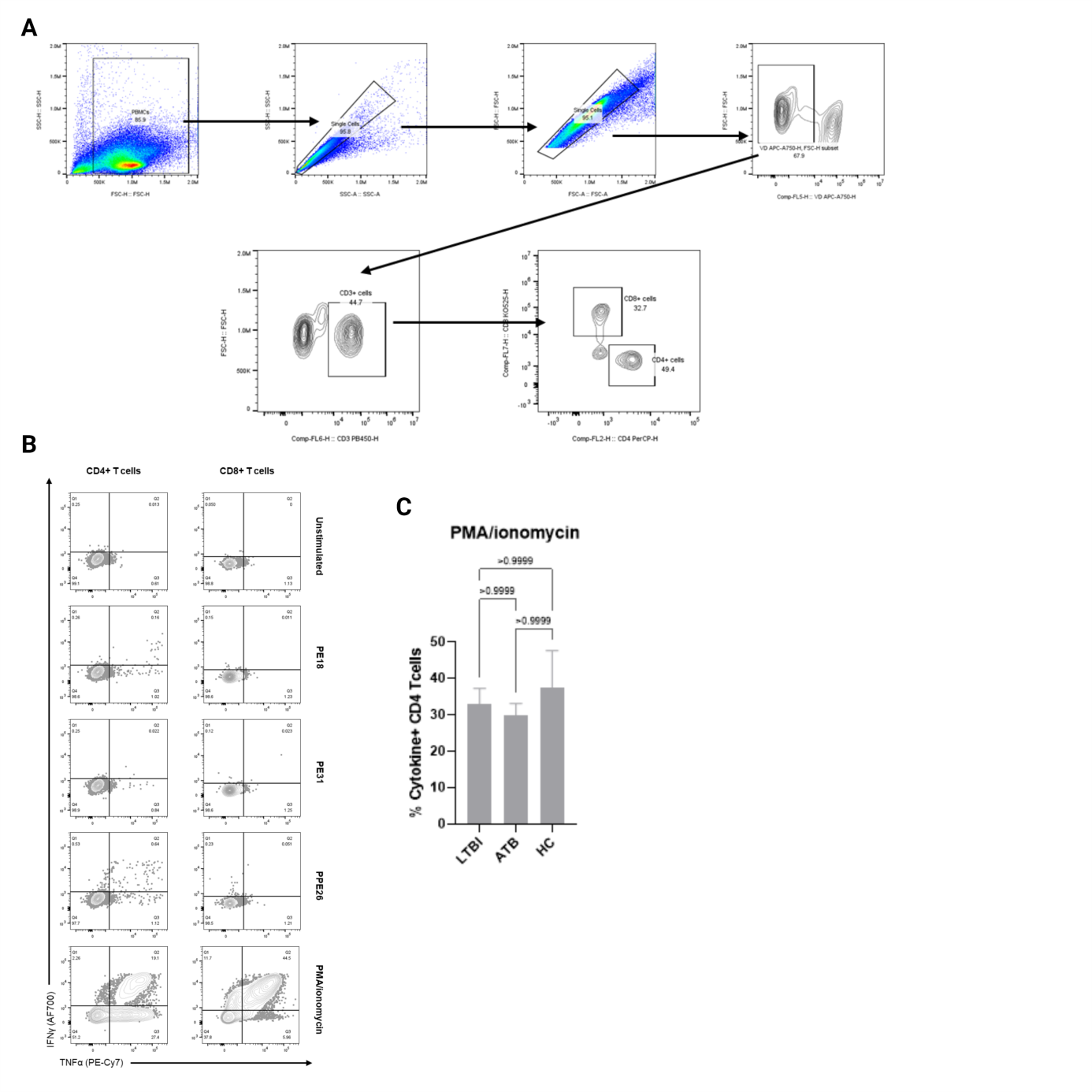


**Supplementary Figure 4.** Gating strategy and representative Th1 cytokine expression in human PBMC from individuals with exposure to mycobacteria. The PBMC were stimulated *in vitro* with respective antigens. **(A)** Gating strategy shows first the selection of single cells based on forward scatter (FSC) and side scatter (SSC). Live cells were further identified, followed by gating on CD3+ cells to select T cells. From the CD3+ population, CD4+ and CD8+ T cells were specifically gated to analyze Th1 cytokine expression. **(B)** Representative dot plot is depicted for one donor, displaying Th1 cytokine (IFNγ and TNF) expression in unstimulated, antigen-stimulated (PE18, PE31, or PPE26), and PMA/ionomycin-stimulated samples. **(C)** The proportion of Th1 cytokine-positive CD4 T cells after PMA/ionomycin treatment of cells from individuals with latent TB infection (LTBI), active TB disease (ATB), and healthy controls (HC, BCG vaccinated) is presented. Data were analyzed using a Kruskal-Wallis test, followed by post hoc Dunn's test to compare each group against the control group (HC). The results are presented as mean with SEM. Significance levels were denoted as numbers.


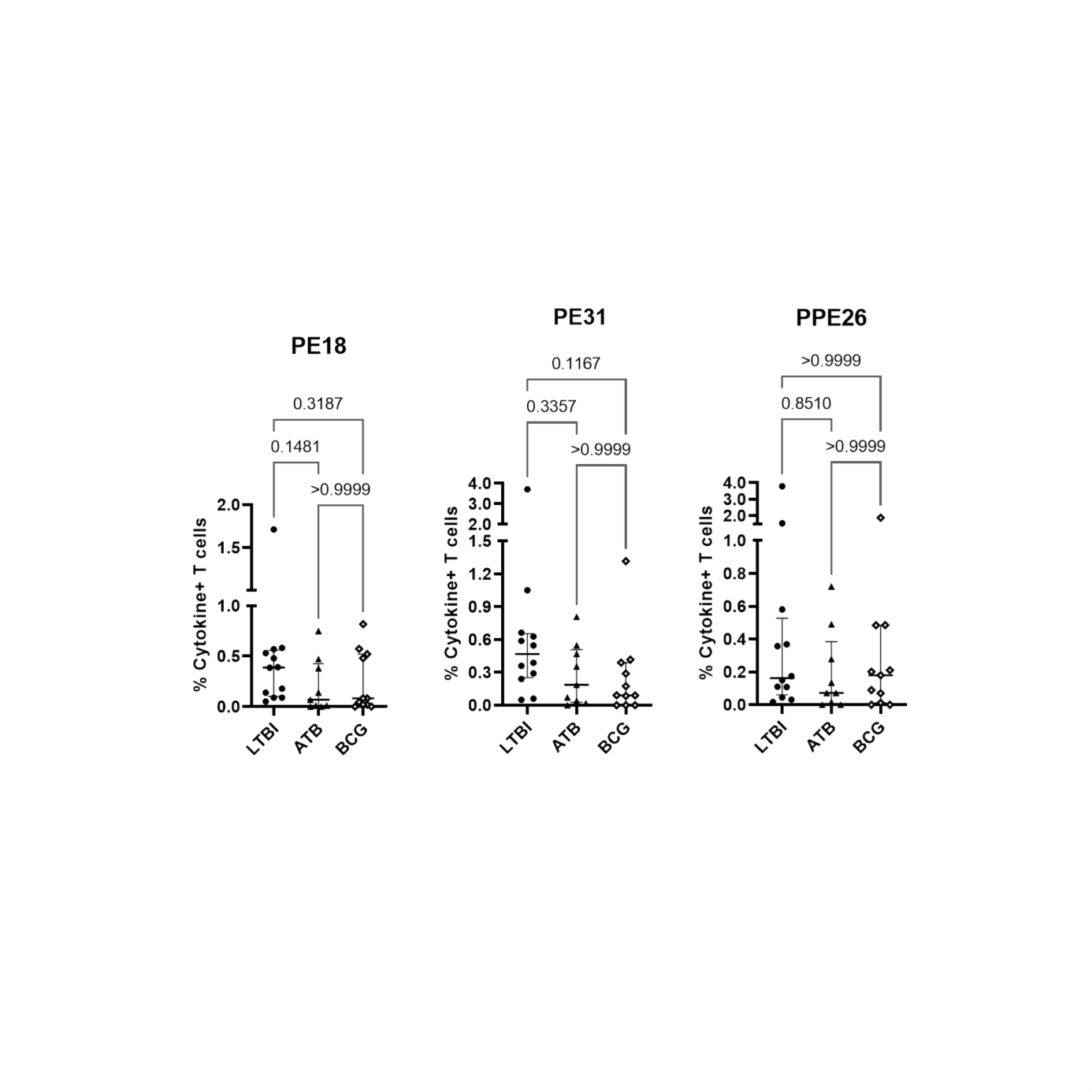


**Supplementary Figure 5.** Antigen-induced T cell proliferation in PBMC from individuals exposed to *Mtb* or BCG. Human PBMCs from individuals with exposure to *Mtb* (ATB or LTBI) or BCG were stimulated *in vitro* with each respective protein. For each antigen, the proportion of all T cells producing IFNγ and TNF is presented for the LTBI, ATB, and BCG groups. The results show the individual values with the median and the interquartile range (IQR) for the percentage of IFNγ and TNF positive antigen-specific T-cells. The data were analyzed using a Kruskal-Wallis test followed by Dunn´s test correction. The significance levels were indicated as numbers.

**
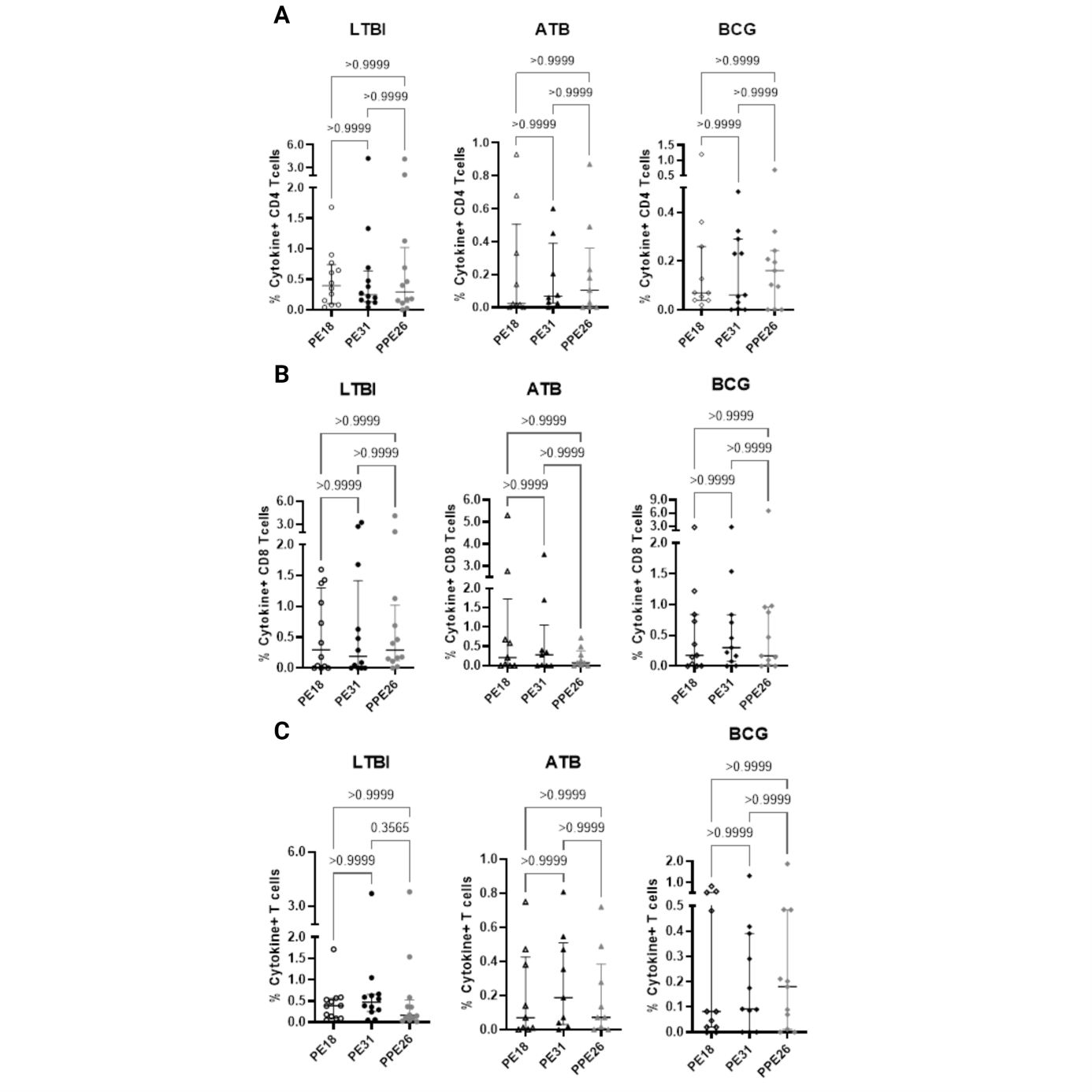
**

**Supplementary Figure 6.** Comparison of PE18, PE31 and PPE26 antigen-specific T cell proliferation responses in LTBI, ATB and BCG-vaccinated individuals. For each sub-cohort, the percentage of IFNγ and TNF positive antigen-specific CD4 **(A),** CD8 **(B)** and total CD4 and CD8 **(C)** T cells for each antigen are represented. The results represent individual values with median and IQR for the percentage of IFNγ and TNF positive antigen-specific T-cells, analyzed using a Kruskal-Wallis analysis followed by Dunn´s test correction. The significance levels were indicated as numbers.

**
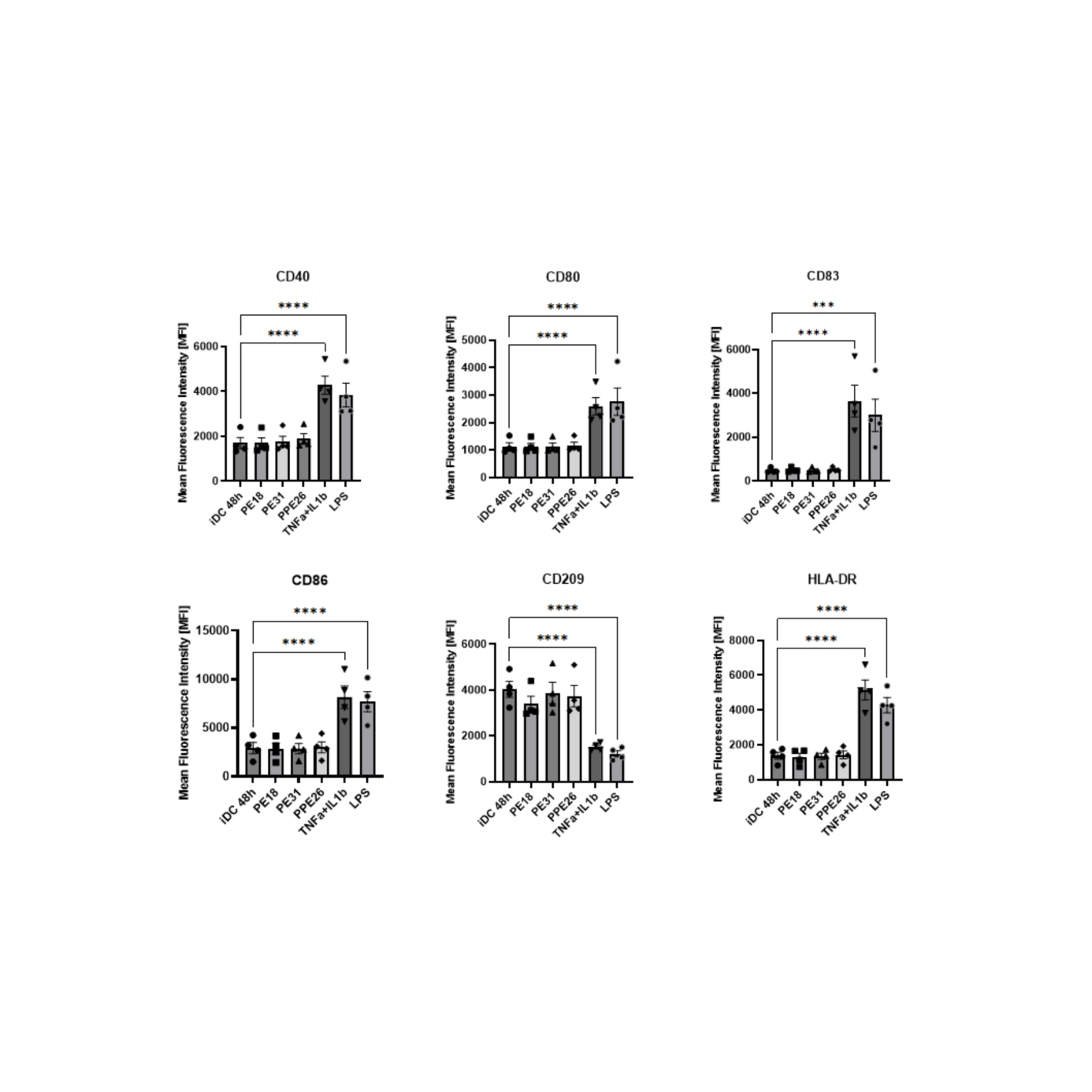
**

**Supplementary Figure 7.** Evaluation of dendritic cell maturation induced by PE/PPE antigens. The assay was performed using dendritic cells (DCs) generated from monocytes derived from peripheral blood mononuclear cells (PBMCs) obtained from healthy human donors with no record of prior exposure to mycobacteria. Initially, the DCs were stained with fluorescence-activated cell sorting (FACS) markers to assess their immature dendritic cell (iDC) state, including CD14, CD40, CD80, CD83, CD86, CD209, and HLA-DR. Subsequently, the respective antigens (at a concentration of 10µg/mL) were added to the cell culture and the cells were incubated for 48 hours. The mature state was assessed by staining with same FACS markers. Lipopolysaccharide (LPS) at a concentration of 1µg/mL was used as a positive control. The resulting fluorescence data were collected and processed using FACS. Statistical comparisons were performed between the experimental groups and the control condition (iDCs). These results depict the mean fluorescence intensity (MFI) for each marker assessed. To assess significant differences between the experimental groups and the control condition, a one-way ANOVA with assumed sphericity was performed, followed by Dunnett correction. The levels of significance are indicated by asterisks as follows: (***, p < 0.001) and (****, p < 0.0001).

**
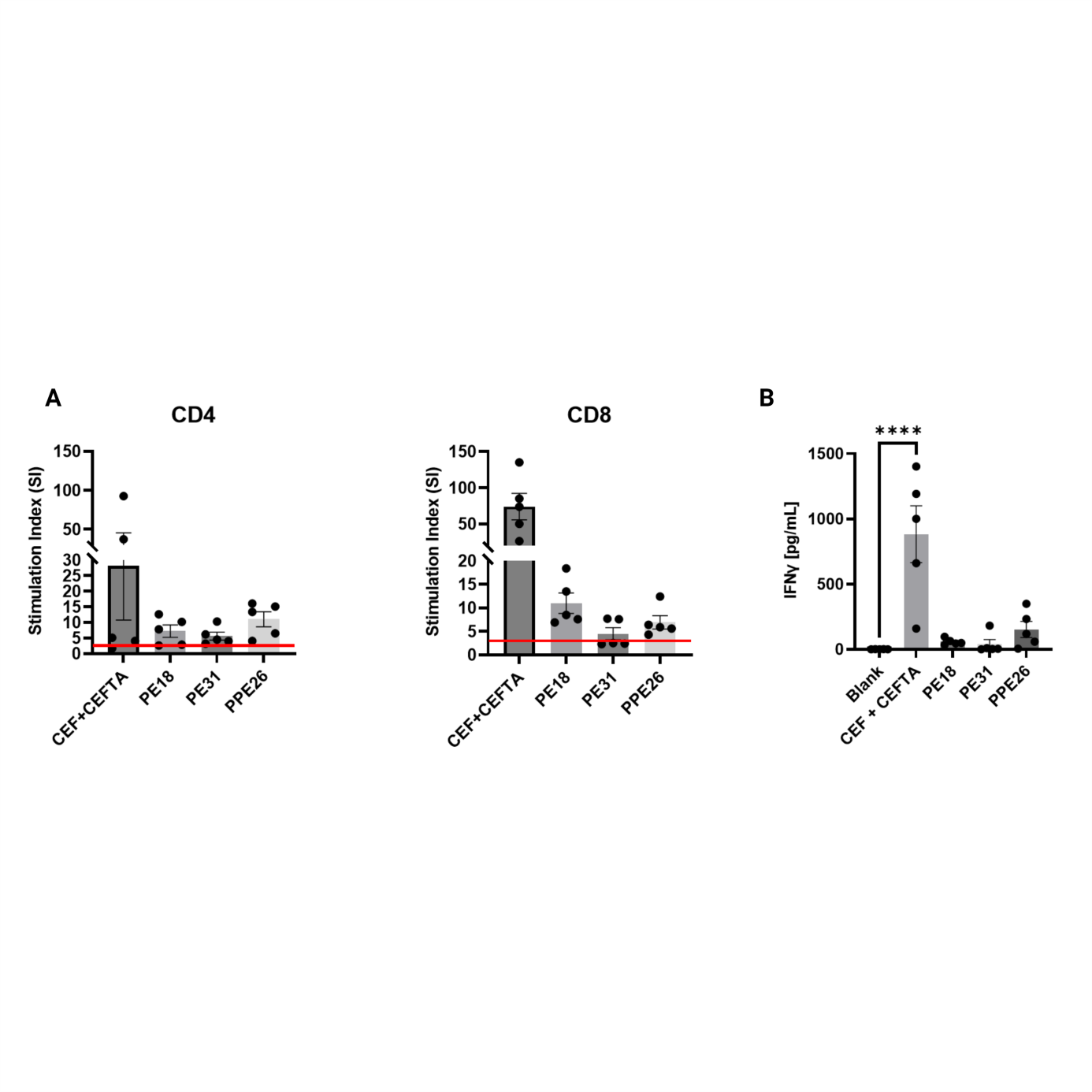
**

**Supplementary Figure 8.** Antigen-induced T cell proliferation in unexposed hosts. Human PBMCs from unexposed healthy donors were stimulated in vitro with each antigen separately at a concentration of 10µg/mL. **(A)** Proliferation of antigen-specific CD4+ and CD8+ T cell populations in five different donors by PPE26, PE18 or PE31 was measured in terms of EdU incorporation using flow cytometry, and calculated as stimulation index (SI). Stimulation index indicates the ratio of the mean response of the test product condition over the mean response of the control condition, and it is considered positive when the response in higher that SI=2. **(B)** Levels of IFNγ production (pg/mL) in the harvested supernatants after 7 days were measured. CEF+CEFTA peptide pools were included as positive control for T cell stimulation. The results represent mean values with SEM of IFNγ levels according to a one-way ANOVA with assumed sphericity, followed by Dunnett test correction (****, p < 0.0001).


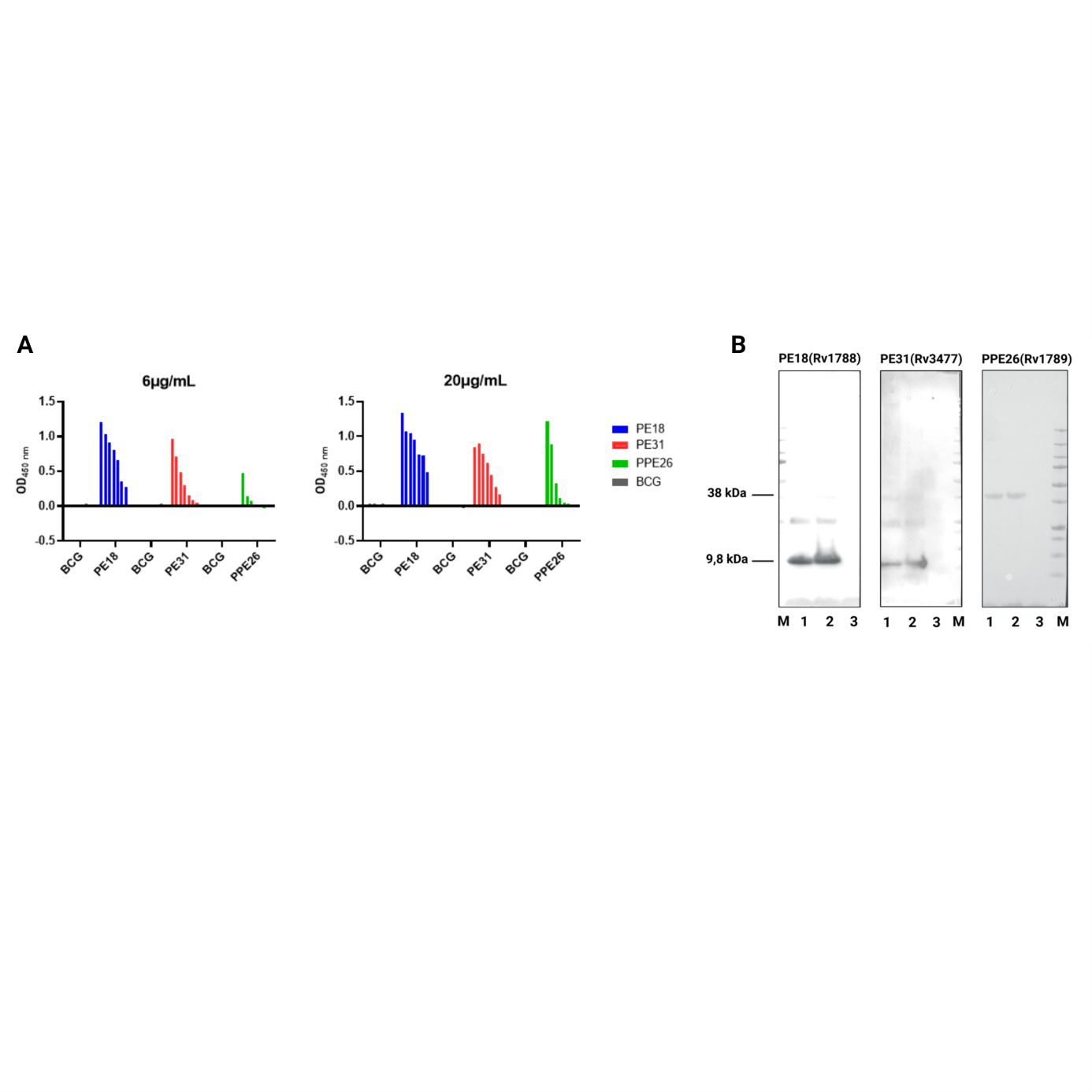


**Supplementary Figure 9.** Reactivity of PPE26, PE18 and PE31 from Mtb H37Rv in BCG Pasteur strain. Sera from immunized mice were evaluated for antigen-specific IgG antibodies against proteins PPE26, PE18 and PE31, as well as the BCG Pasteur strain, using ELISA and western blot analysis. For ELISA **(A)**, two different concentrations (6 and 20µg/mL) of each antigen or BCG were coated on the ELISA plates. The absorbance at 450nm was measured to assess the antibody response. Results are presented as the signal of serial dilutions, reflecting the antibody reactivity. **(B)** Proteins were subjected to electrophoresis under reducing and non-reducing conditions, followed by transfer onto a PVDF membrane, along with BCG Pasteur strain. The membranes were probed with sera from immunized mice to detect the reactivity or presence of these proteins in the BCG strain. Lane 1 corresponds to non-reducing conditions, Lane 2 to reducing conditions, and Lane 3 to BCG lysate. The molecular sizes of the individual antigens are indicated in bold. The ladder/marker (M) was included for size reference.
